# Open chromatin interaction maps reveal functional regulatory elements and chromatin architecture variations during wheat evolution

**DOI:** 10.1186/s13059-022-02611-3

**Published:** 2022-01-24

**Authors:** Jingya Yuan, Haojie Sun, Yijin Wang, Lulu Li, Shiting Chen, Wu Jiao, Guanghong Jia, Longfei Wang, Junrong Mao, Zhongfu Ni, Xiue Wang, Qingxin Song

**Affiliations:** 1 State Key Laboratory of Crop Genetics and Germplasm Enhancement, Jiangsu Collaborative Innovation Center for Modern Crop Production, Nanjing Agricultural University, No. 1 Weigang, Nanjing, 210095 Jiangsu China; 2 State Key Laboratory for Agrobiotechnology, Key Laboratory of Crop Heterosis and Utilization (MOE), Beijing Key Laboratory of Crop Genetic Improvement, China Agricultural University, Beijing, 100193 China

**Keywords:** Open chromatin interaction, Wheat, Polyploidy, Evolution, OCEAN-C

## Abstract

**Background:**

Bread wheat (*Triticum aestivum*) is an allohexaploid that is generated by two subsequent allopolyploidization events. The large genome size (16 Gb) and polyploid complexity impede our understanding of how regulatory elements and their interactions shape chromatin structure and gene expression in wheat. The open chromatin enrichment and network Hi-C (OCEAN-C) is a powerful antibody-independent method to detect chromatin interactions between open chromatin regions throughout the genome.

**Results:**

Here we generate open chromatin interaction maps for hexaploid wheat and its tetraploid and diploid relatives using OCEAN-C. The anchors of chromatin loops show high chromatin accessibility and are concomitant with several active histone modifications, with 67% of them interacting with multiple loci. Binding motifs of various transcription factors are significantly enriched in the hubs of open chromatin interactions (HOCIs). The genes linked by HOCIs represent higher expression level and lower coefficient expression variance than the genes linked by other loops, which suggests HOCIs may coordinate co-expression of linked genes. Thousands of interchromosomal loops are identified, while limited interchromosomal loops (0.4%) are identified between homoeologous genes in hexaploid wheat. Moreover, we find structure variations contribute to chromatin interaction divergence of homoeologs and chromatin topology changes between different wheat species. The genes with discrepant chromatin interactions show expression alteration in hexaploid wheat compared with its tetraploid and diploid relatives.

**Conclusions:**

Our results reveal open chromatin interactions in different wheat species, which provide new insights into the role of open chromatin interactions in gene expression during the evolution of polyploid wheat.

**Supplementary Information:**

The online version contains supplementary material available at 10.1186/s13059-022-02611-3.

## Background

The interplay of *trans*-acting factors and *cis*-regulatory elements (CREs) orchestrates temporal and spatial patterns of gene expression in plant development and environmental response [1, 2]. Actively engaged CREs normally reside in accessible chromatin regions (ACRs) [3]. Several methods including ATAC-seq and DNase-seq are developed to identify open chromatin regions throughout the genome [4, 5]. Genetic variation in CREs such as promoters, enhancers, and insulators can lead to expression changes of linked genes and corresponding morphological variations. For example, a single nucleotide polymorphism variation at 1818 bp upstream of *TGW2* induces its expression change and thus alters grain width and weight by influencing cell proliferation and expansion in glumes [6]. The three-dimensional (3D) folding of the eukaryotic genome brings long-range interactions between genomic elements that are tightly linked to gene expression [7, 8]. Recent advances in chromosome conformation capture (3C)-based methods, including Hi-C and ChIA-PET, have provided comprehensive long-range chromatin interaction maps in animals [9, 10] and plants [8, 11–14]. Extensive chromatin interactions could occur between genes and genes, or between genes and distal regulatory elements. The genes with chromatin loops normally show higher expression levels than genes without loops [8, 15]. The dynamics of chromatin loops are involved in organ development and stress response in *Arabidopsis*, rice, and other plants [14, 16]. In maize, a hepta-repeat located ~ 100 kb upstream of *BOOSTER1* (*B1*) physically interacts with its transcription start region to modulate anthocyanin biosynthesis by regulating *B1* expression [17, 18].

Polyploidy is widespread in plants and more than 70% of angiosperms are considered to be polyploids [19]. Polyploidy causes dramatic chromosomal rearrangement and epigenetic changes, leading to alteration of transcriptome networks [20]. Wheat is a powerful model for studying chromosome topology and genetic interactions between subgenomes in polyploids. Bread wheat (*Triticum aestivum*) is a widely cultivated allohexaploid crop and evolved through two rounds of interspecific hybridization and polyploidization. The first allotetraploidization occurred 0.36 to 0.50 million years ago and involved hybridization between *Triticum urartu* (AA) and an undiscovered or extinct species closely related to the *Aegilops speltoides* (SS) [21]. The second allohexaploidization between tetraploid wheat (*Triticum turgidum L.*, AABB) and a goatgrass species (*Aegilops tauschii*, DD) ~ 8000–10,000 years ago resulted in the formation of bread wheat [22].

Recent researches about wheat chromosome architecture using Hi-C and HiChIP represented the presence of subgenome-specific territories and highly coordinated expression of genes involved in RNA polymerase II-associated loops [23, 24]. Yet important questions remain unanswered, such as how the distal CREs regulate gene expression, whether chromatin interactions are involved in expression bias of homoeologous genes and how chromatin loops contribute to polyploid wheat evolution. Wheat has a large complex genome (~ 16 Gb) with ~ 85% transposable elements (TEs) [25]. Hi-C samples proximity ligations between all possible pairs of fragments indiscriminately and thus requires billions of reads to achieve truly genome-scale coverage at kilobase-pair resolution for large genomes [26]. HiChIP provides robust loop calling with low sequencing depth, however, HiChIP is antibody-dependent and only captures the subset of chromatin interactions mediated by a specific protein of interest. How to identify chromatin loops at high resolution and low sequencing costs is still a challenge for wheat.

In this study, we generated chromatin interaction maps for hexaploid wheat and its tetraploid and diploid relatives using open chromatin enrichment and network Hi-C (OCEAN-C), which could capture global chromatin interactions between open chromatin regions without relying on specific antibodies [27]. By integrating OCEAN-C, ChIP-seq, ATAC-seq, and RNA-seq data, we revealed open chromatin interactions and their relationship with epigenetic marks, chromatin accessibility, and gene expression in wheat. We found enrichment of various transcription factor (TF) binding motifs in hubs of open chromatin interactions, which could coordinate co-expression of linked genes. Additionally, we demonstrated the spatial conformation reorganization contributes to expression variation between hexaploid wheat and its tetraploid and diploid relatives. These results provide new insights into the role of open chromatin interactions in gene expression during the evolution of polyploid wheat.

## Results

### Genome-wide open chromatin interactions in hexaploid wheat

**Fig. 1:**
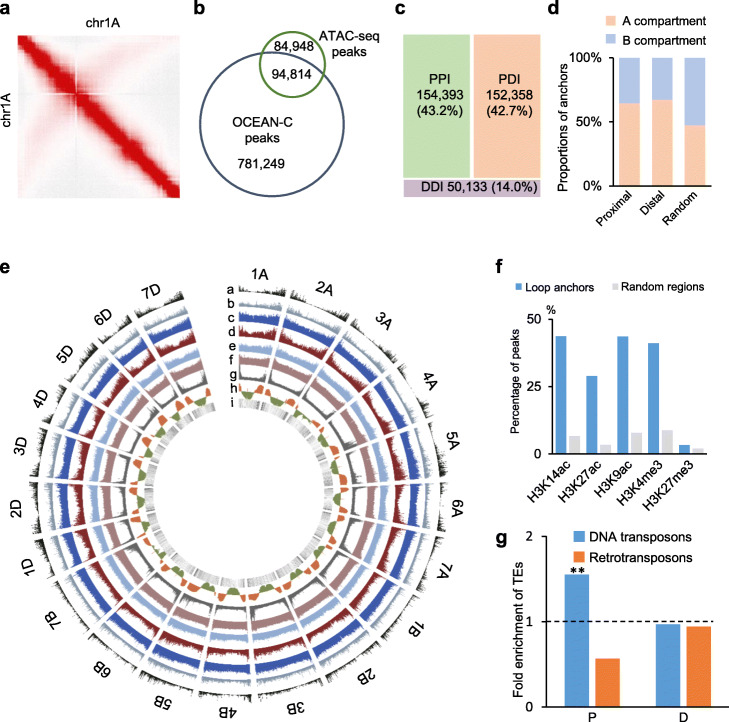
Distribution of OCEAN-C loops across wheat genome. **a** OCEAN-C interaction matrix at 1 Mb resolution throughout chromosome 1A. **b** Venn diagram showing the overlapped peaks of OCEAN-C and ATAC-seq. **c** Fractions of PPI (green), PDI (orange), and DDI (purple) loops in all identified OCEAN-C loops. **d** Proportions of proximal and distal peaks in A/B compartments. The windows at 5 kb were randomly selected as random anchors using BEDTools from each chromosome. **e** Distribution of OCEAN-C anchors (a), chromatin accessibility (b), H3K9ac (c), H3K14ac (d), H3K27ac (e), H3K4me3 (f), H3K27me3 (g), A/B compartments (h), and gene density (i) throughout chromosomes in the hexaploid wheat. **f** Fractions of OCEAN-C loop anchors overlapped with various histone modification markers and open chromatin regions identified by ATAC-seq. Randomly selected regions were used as control. **g** Fold-enrichment of TEs in proximal (P) and distal (D) anchors, relative to whole genome. ** indicates *P* < 0.01 (Hypergeometric test)

To investigate global open chromatin interactions in wheat, we performed OCEAN-C experiments with two biological replicates using young leaves of hexaploid *T. aestivum* cv. Chinese Spring (AABBDD). A total of 276 million valid interaction pairs were obtained and high reproducibility was observed between two biological replicates (Pearson correlation = 0.97) (Additional file 1: Table S1). We also performed ATAC-seq for chromatin accessibility, ChIP-seq for histone modifications, and RNA-seq for gene expression to further analyze the relationships between open chromatin interactions, epigenetic marks, chromatin accessibilities and gene expression (Additional file 1: Table S2 and Table S3). There was high reproducibility between biological replicates (Pearson correlation = 0.95~0.99) for ATAC-seq, ChIP-seq, and RNA-seq (Additional file 1: Table S2), and 56~ 95% peaks were overlapped between two biological replicates (Additional file 2: Fig. S1a). The OCEAN-C interaction map at the chromosome level showed strong signals along the main diagonals and less prominent signals on the anti-diagonal lines (Fig. 1a and Additional file 2: Fig. S2a). This conformation reflects Rabl chromosome configuration and is similar with the Hi-C interaction matrixes constructed using uniquely mapped reads in recent studies [23, 24], indicating OCEAN-C data could represent high-order organization of wheat genome at a low resolution in addition to capturing open chromatin loops.

As nucleosome-depleted chromatin regions are enriched in procedures of the OCEAN-C experiment, we first identified 891,128 high-confidence OCEAN-C peaks with an average length of 4.6 kb, which stand for anchors of open chromatin loops. More than half (53%) of ATAC-seq peaks were overlapped with OCEAN-C peaks (Fig. 1b), confirming that these peaks are open chromatin regions. Next, we identified 356,884 intrachromosomal loops that connect these OCEAN-C peaks (Additional file 3: Table S4). To verify whether OCEAN-C loops could capture chromatin loops between open chromatin regions, we re-analyzed published RNA Pol II HiChIP data using the same read mapping and loop calling strategies of OCEAN-C analysis [24]. In total, 50% of RNA Pol II peaks were overlapped with OCEAN-C peaks (Additional file 2: Fig. S2b). And 73.5% of HiChIP loops were overlapped with OCEAN-C loops (Additional file 2: Fig. S2c), suggesting OCEAN-C loops could capture most of RNA Pol II HiChIP loops.

The peaks within 3 kb around genes were annotated as proximal peaks (P) and the peaks located more than 3 kb away from genes were annotated as distal peaks (D). The intrachromosomal loops contained 154,393 (43.2%) P-P interactions (PPI), 152,358 (42.7%) P-D interactions (PDI), and 50,133 (14.0%) D-D interactions (DDI) (Fig. 1c). These loop anchors were predominately distributed in distal ends of chromosomes and mainly enriched in A compartments (Fig. 1d, e). The loop anchors were enriched in accessible chromatin regions and mainly occupied by active histone modifications peaks but not the repressive histone modification H3K27me3 (Fig. 1f). Furthermore, DNA transposable elements (TEs) were enriched in proximal OCEAN-C anchors but not in distal OCEAN-C anchors (Fig. 1 g).

**Fig. 2:**
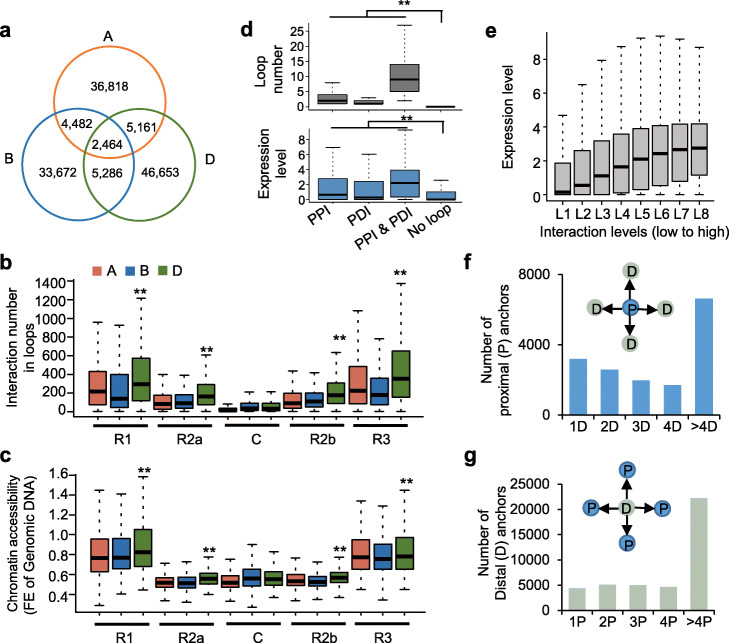
Higher expression level in genes with more chromatin interactions. **a** Venn diagram showing the homoeologous PPI overlap between A, B, and D subgenomes. **b, c** Distribution of chromatin interactions (**b**) and chromatin accessibility (**c**) in distal telomeric regions (short arm, R1; long arm, R3), interstitial regions (short arm, R2a; long arm, R2b), and centromere regions (C) of chromosomes. Chromosomes were divided into 1 Mb windows. ** indicates *P* < 0.01 (Wilcoxon rank-sum test). **d** The loop numbers (top) and expression levels (bottom, log_2_(FPKM+ 1)) of genes with different types of OCEAN-C loops and without loop. ** indicates *P* < 0.01 (Wilcoxon rank-sum test). **e** Expression levels (log_2_(FPKM+ 1)) of genes with different levels of chromatin interactions. **f** The distribution of proximal anchors interacting with various numbers of distal anchors. **g** The distribution of distal anchors interacting with various numbers of proximal anchors

The A, B, and D subgenomes respectively contained 48,925, 45,904, and 59,564 PPIs, of which only 2464 PPIs were conserved among all three subgenomes (Fig. 2a). These results suggest that asymmetrical interactions widely occur between subgenomes in wheat. Interestingly, there were significantly more open chromatin loops in chromosomal ends of D subgenome compared with A and B subgenomes (Fig. 2b, *P* < 0.01), which may be due to higher chromatin accessibility in chromatin ends of D subgenome (Fig. 2c) [28]. Chromatin loops are observed to be involved in expression regulation in previous reports [15, 29, 30]. In wheat, the genes with loops, especially with both PPI and PDI, showed much higher expression levels than genes without loops (Wilcoxon rank-sum test, *P* < 0.01) (Fig. 2d). When genes were divided to eight groups according to chromatin interaction numbers, the genes with more chromatin interactions displayed higher expression levels (Fig. 2e). These results indicate positive correlation between chromatin interaction density and expression levels of genes.

### Hubs of open chromatin interactions in wheat genome

**Fig. 3:**
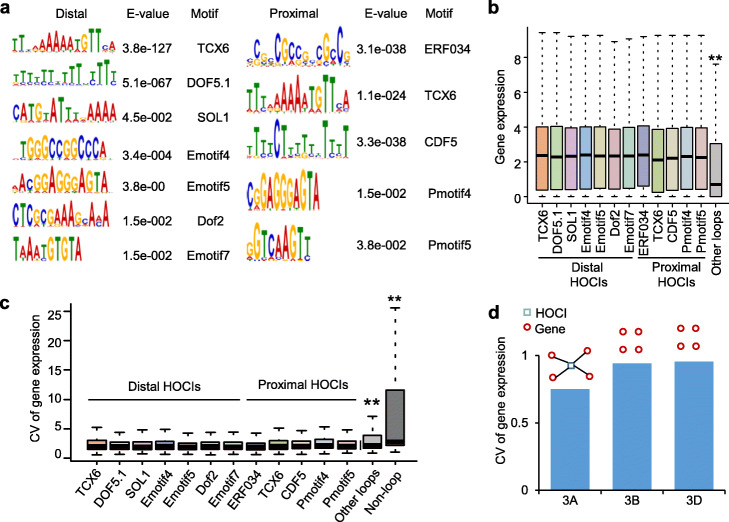
Coordinated expression of genes linked by HOCIs. **a** TF binding motifs enriched in the distal (left) and proximal (right) HOCIs. **b** Expression values (log_2_(FPKM+ 1)) of the genes that interacted with TF-binding motif enriched distal and proximal HOCIs. The genes interacted with other loci were used as control. ** indicates *P* < 0.01 (Wilcoxon rank-sum test). **c** Counterpart genes of proximal and distal HOCIs showing significantly lower coefficient of expression variance (CV) than the genes linked by other loops or the genes without loop. ** indicates *P* < 0.01 (Wilcoxon rank-sum test). **d** An example of the genes that interacted with a same distal HOCl showing lower CV than the homoeologous genes which were not linked by a single HOCI

We found 41.2% of proximal anchors interacted with more than 4 distal loci (Fig. 2f). Accordingly, 67.7% of distal anchors interacted with more than 4 proximal loci (Fig. 2 g). The anchors that interacted with multiple loci showed more active histone modifications and higher chromatin accessibility than anchors that interacted with only one locus (Additional file 2: Fig. S3). The proximal and distal anchors that interacted with more than 4 loci were defined as proximal and distal hubs of open chromatin interactions (HOCIs). We found binding motifs of various transcription factors, including Ethylene-Response Factors (ERFs), DNA-binding with one finger (DOF), and floral organ development factors, were over-represented in proximal and distal HOCIs (Fig. 3a). The genes that interacted with TF-binding motif enriched HOCIs showed significantly higher expression levels than the genes linked by other loops (*P* < 0.01, Wilcoxon rank-sum test) (Fig. 3b). Strikingly, the genes linked by the same distal and proximal HOCIs showed significantly lower coefficient of expression variance (CV) than the genes with other loops and genes without loops (*P* < 0.01, Wilcoxon rank-sum test) (Fig. 3c). For example, four genes (*TraesCS3A01G204900*, *TraesCS3A01G205300*, *TraesCS3A01G205100*, and *TraesCS3A01G205600*) interacted with a same HOCI in chromosome 3A showed lower CV than the homoeologous genes in chromosome 3B and 3D that were not looped by a specific region (Fig. 3d). Our results suggest that HOCIs may coordinate co-expression of linked genes.

Furthermore, we identified 1301 chromatin interaction networks (ChINs) containing at least three chromatin loop anchors (Additional file 3: Table S4), of which 920 ChINs had more than 30 nodes. We proposed that genes connected in the ChINs were prone to function in related biological processes. To validate this, we analyzed the enrichment of GO terms for the top 10 ChINs. We found the enrichment of many GO terms in ChINs, such as nucleotide binding, transcription factor activity, carbohydrate binding, and protein modification process (Additional file 2: Fig. S4a). For example, a ChIN on chromosome 5A contained three genes that were involved in the regulation of photoperiodic flowering [31–33] (Additional file 2: Fig. S4b).

### Structural variations contribute to interaction divergence between subgenomes

**Fig. 4:**
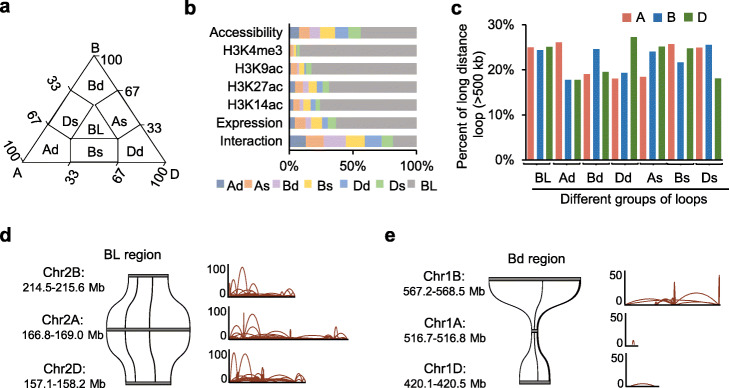
Chromatin interaction bias of syntenic homoeolog triads in wheat. **a** Seven types of triads (A, B, and D homoeologs) showing balanced (BL) and unbalanced (A dominant: Ad; B dominant: Bd; D dominant: Dd; A suppressed: As; B suppressed: Bs; D suppressed: Ds) chromatin interactions among homoeologs. **b** Percentages of balanced and unbalanced triads for gene expression, chromatin interaction, chromatin accessibility and histone modifications. **c** Percentages of long distance loops (more than 500 kb) overlapping with homoeologs in balanced and unbalanced triads. **d, e** Examples showing the collinearity (left panel) and chromatin interactions (right panel) in genomic regions containing balanced (**d**) or B dominant (**e**) homoeolog traids

Although many genes exist in triplicate in wheat genome, transcriptional divergence between homoeologous genes is widely observed in hexaploid wheat [34]. To examine the effects of epigenetic marks and chromatin interactions on homoeolog expression divergence, we identified 16,783 genes in triads (1:1:1 correspondence across the three homoeologous subgenomes) in wheat genome. We divided all triads into seven groups according to divergence between A, B, and D homoeologs: a balanced group (BL), three subgenome dominant groups (Ad, Bd, Dd) and three subgenome suppressed groups (As, Bs, Ds) (Fig. 4a). Most triads showed the balance of expression levels and histone modifications (Fig. 4b). However, chromatin interaction and chromatin accessibility were highly divergent among homoeologous genes (Fig. 4b and Additional file 4: Table S5). Unexpectedly, the homoeologous genes with a significant divergence of chromatin interactions did not show obvious bias of chromatin accessibility (Additional file 2: Fig. S5a), suggesting interaction divergence of homoeologous genes were not due to chromatin accessibility bias. Furthermore, gene expression bias was also not observed in homoeologous genes with chromatin interaction divergence (Additional file 2: Fig. S5b).

The chromatin loops that linked homoeologous genes with balanced chromatin interactions showed a similar fraction of long distance loop (> 500 kb) among subgenomes (Fig. 4c). For example, genomic regions containing homoeologous genes with balanced chromatin interactions displayed similar genomic length and good collinearity among subgenomes (Fig. 4d). Whereas the chromatin loops that linked genes with dominant chromatin interactions showed a higher fraction of long distance loop than the chromatin loops that linked counterpart homoeologs (Fig. 4c). In contrast, the chromatin loops that linked genes with suppressed chromatin interactions showed lower fraction of long distance loop (Fig. 4c). For example, genomic region in B subgenome (Chr1B: 567.2–568.5 Mb) containing genes with Bd chromatin interactions was much larger than homoeologous genome regions in A (Chr1A: 516.7–516.8 Mb) and D subgenome (Chr1D:420.1–420.5 Mb) (Fig. 4e). These results suggested sequence insertion/deletion or structural variation in intergenic regions may induce generation of long distance loop (> 500 kb) to influence interaction divergence among subgenomes.

### Interchromosomal interactions in wheat genome

**Fig. 5:**
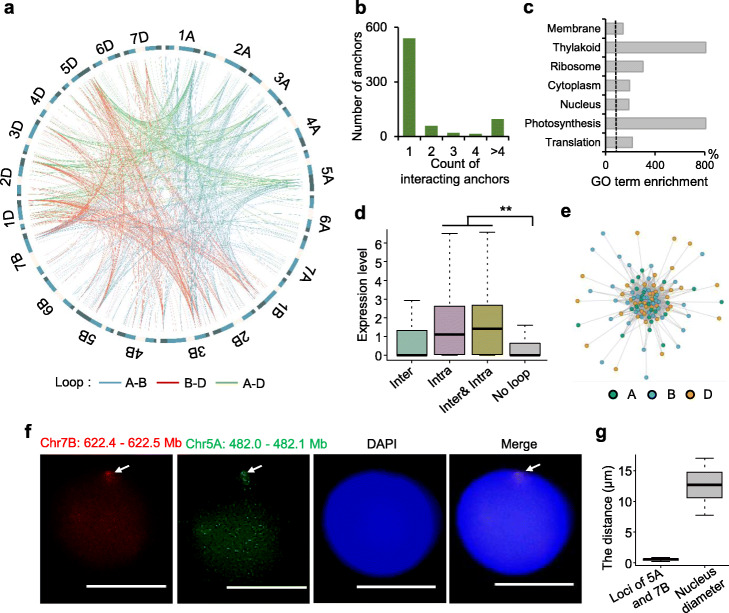
Interchromosomal loops in wheat genome. **a** Circos plot of interchromosomal loops in wheat genome. The loops between different subgenomes were in different colors (A–B: blue; A–D: green; B–D: red). **b** The distribution of anchors interacting with various numbers of loci. **c** Significantly enriched GO terms for genes with interchromosomal loops (hypergeometric test, *P* < 0.05). **d** The expression levels (log_2_(FPKM+ 1)) of genes only with interchromosomal loops (Inter), genes only with intrachromosomal loops (Intra), genes with both inter- and intra-chromosomal loops and genes without loops. **e** An interchromosomal interaction network that was formed by interactions among A (green), B (blue), and D (yellow) subgenomes. **f** 3D-FISH showing spatial proximity of one locus (Chr5A: 482–482.1 Mb) and another locus (Chr7B: 622.4–622.5 Mb) in nucleus of five-leaf stage leaves. The exons in these two regions were used as probes labeled with different colors (Chr5A: 482–482.1 Mb: Fluorescein-12-dUTP; Chr7B: 622.4–622.5 Mb: Texas Red-12-dUTP). Scale bars = 10 μm. g. The distance distribution of two loci (Chr5A: 482–482.1 Mb; Chr7B: 622.4–622.5 Mb) in 50 leaf nuclei. The nucleus diameter was used as control

Similar with previous reports, interchromosomal interactions occurred at a much lower frequency than intrachromosomal interactions (Additional file 2: Fig. S2a). To explore the potential roles of interchromosomal loops, a total of 1612 interchromosomal loops were identified (Additional file 5: Table S6). The interchromosomal loops linked 729 anchors and included 1074 loops between different subgenomes (407 for A–B, 299 for A–D, and 368 for B–D) (Fig. 5a). Previous wheat Hi-C data showed that interchromosomal interactions mainly occurred in homoeologous genomic regions [24]. However, we found rare interchromosomal loops (0.4%) linked homoeologous genes. About 75% of anchors interacted with only one locus in other chromosomes (Fig. 5b). Interestingly, similar to soybean [15], the interchromosomal loops were enriched in photosynthesis and translation-related terms (Fig. 5c). The genes linked only by intrachromosomal loops and the genes with both inter- and intra-chromosomal loops displayed significantly higher expression levels than genes without loops (*P* < 0.01, Wilcoxon rank-sum test) (Fig. 5d), suggesting both inter- and intra- chromosomal loops were positively associated with gene expression. We identified 19 interchromosomal interaction networks consisting of at least 3 loop anchors. For example, 23, 42, and 39 anchors in A, B, and D subgenomes could be linked with each other to establish an interchromosomal interaction network (Fig. 5e). To further validate interchromosomal interactions, we performed 3D-FISH to examine an interchromosomal loop connecting two anchors, in which one anchor was located in Chr5A (482.0–482.1 Mb) and another anchor was located in Chr7B (622.4–622.5 Mb) (Fig. 5f,g). The average distance of these two loci was 0.55 μm compared with 13 μm nucleus diameter, suggesting that these two anchors were spatially proximate although they were located in different chromosomes (Fig. 5f,g).

### Genetic variants altered chromatin topology and gene transcription during wheat evolution

**Fig. 6:**
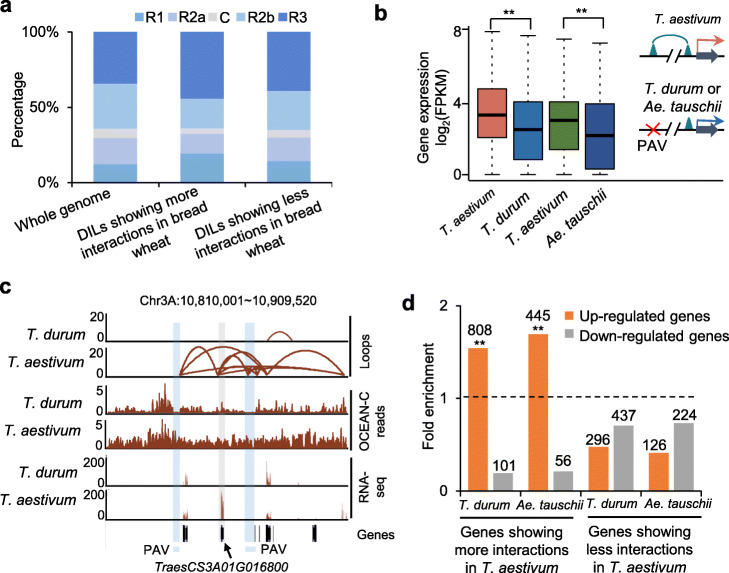
The effects of chromatin topology variations on transcription during wheat evolution. **a** The distribution of differentially interacted loops (DILs) between common wheat and its tetraploid and diploid relatives in distal telomeric regions (short arm, R1; long arm, R3), interstitial regions (short arm, R2a; long arm, R2b), and centromere regions (C) of chromosomes. **b** Transcription levels of genes with and without loops in *T. aestivum* and its tetraploid and diploid relatives that were caused by PAV. ** indicates *P* < 0.01 (Wilcoxon rank-sum test). **c** An example showing chromatin loops present in *T. aestivum* but absent in *T. durum* that were caused by PAVs. **d** Fold-enrichment of differentially expressed genes (DEGs) in the genes overlapping with differential chromatin interactions between *T. aestivum* and its tetraploid and diploid relatives, relative to all expressed genes. ** indicates *P* < 0.01 (hypergeometric test)

To uncover the roles of chromatin loops in transcription regulation during polyploidization in wheat, we further performed OCEAN-C using young leaves of tetraploid *T. turgidum* ssp. *durum* (AABB) and diploid *Ae. tauschii* (DD) in addition to hexaploid *T. aestivum*. OCEAN-C reads of diploid and tetraploid wheat were firstly mapped to published genome sequences of diploid and tetraploid wheat, respectively. However, we found low mapping rates of OCEAN-C data and many anomalous chromatin interaction structures in the OCEAN-C interaction matrix for *Ae. tauschii* (Additional file 2: Fig. S6a-e, Additional file 1: Table S7) [35]. To reduce the effects of genome assembly errors on the analysis of chromatin interactions, we re-mapped OCEAN-C data of the *T. durum* and *Ae. tauschii* to subgenomes of *T. aestivum*, which showed better interaction matrix for *Ae. tauschii* (Additional file 2: Fig. S6f)*.* The Pearson’s correlation coefficient of OCEAN-C data between two biological replicates of *Ae. tauschii* and *T. durum* were 0.99 and 0.98, respectively. A total of 102,266~129,040 intra-loops were identified in each replication of *T. durum* and *Ae. tauschii*, respectively (Additional file 2: Fig. S1b). About 59~ 61% of loops were overlapped between two replications (Additional file 2: Fig. S1b). By comparing *T. aestivum, T. durum*, and *Ae. tauschii*, we identified 21,295 and 20,767 differentially interacted loops (DILs) in AB (*T. aestivum* vs *T. durum*) and D subgenomes (*T. aestivum* vs *Ae. tauschii*), respectively (Additional file 6: Table S8 and Additional file 7: Table S9). The DILs showing more interactions in hexaploid wheat were significantly enriched in distal R1 and R3 chromosome regions (*P* < 0.01, Hypergeometric distribution) (Fig. 6a). To explore the effects of genetic variations on chromatin topology, we identified 94,285 presence/absence variations (PAVs) among different wheat species. We found 3.3% of PAVs overlapped with anchors of DILs. We further identified 1396 PAV-associated loops that were detected in *T. aestivum* but absent in *T. durum* or *Ae. tauschii*. Interestingly, the expression levels of genes linked by these loops in *T. aestivum* were significantly higher than orthologous genes without loops in *T. durum* and *Ae. tauschii* (Fig. 6b). For example, the *TraesCS3A01G016800* gene interacted with two genomic regions which were present in *T. aestivum* but absent in *T. durum* showed higher expression levels in *T. aestivum* (Fig. 6c). To further examine the role of chromatin interaction variations on expression changes during wheat evolution, we identified 4949 and 2804 genes showing significant chromatin interaction differences among different wheat species in AB (*T. aestivum* vs *T. durum*) and D subgenomes (*T. aestivum* vs *Ae. tauschii*), respectively. It is worth noting that up-regulated genes were significantly enriched in genes with more interactions in *T. aestivum* (Fig. 6d). These results indicated that genetic variations could induce changes of chromatin topology and transcription levels of corresponding genes during wheat evolution.

**Fig. 7:**
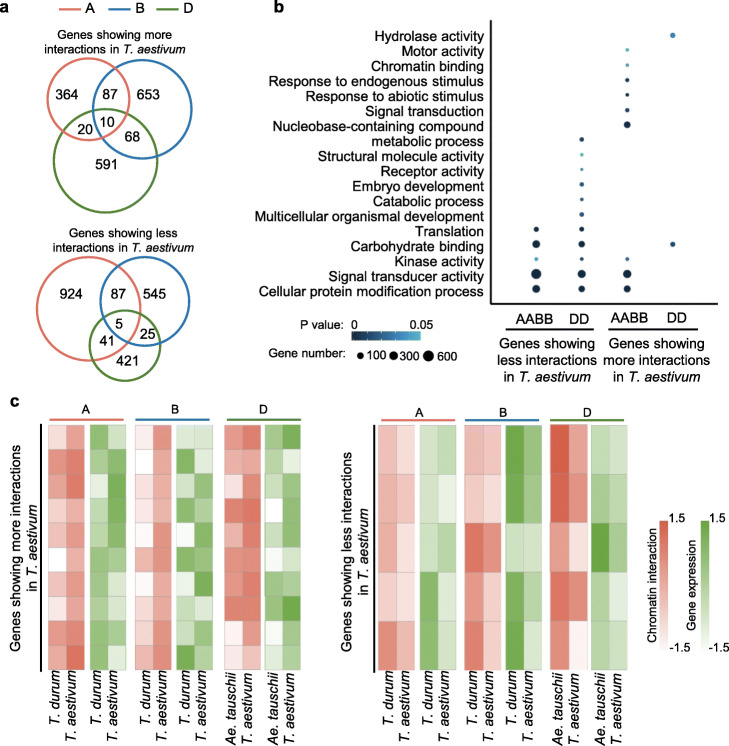
Limited conservation of chromatin interaction changes in homoeolog triads during wheat evolution. **a** The overlap of homoeologs with differentially intrachromosomal loops between common wheat and its tetraploid and diploid relatives. **b** GO analysis of genes overlapping DILs between common wheat and its tetraploid and diploid relatives. **c** The relationship between chromatin interaction intensity and expression levels for homoeolog triads that overlapped DILs in all three homoeologs

For genes in triads, we respectively identified 481, 818, and 689 genes showing more chromatin interactions in A, B, and D subgenomes of *T. aestivum* compared with *T. durum* and *Ae. tauschii* (Fig. 7a). Meanwhile, 1057, 662 and 492 genes contained less chromatin interactions in A, B and D subgenomes of *T. aestivum* (Fig. 7a). These genes in AB and D subgenomes participated in different biological processes. The genes in AB subgenomes were involved in chromatin binding, response to abiotic stimulus, and signal transduction (Fig. 7b), whereas the genes in D subgenome were over-represented in translation, embryo development, and catabolic process (Fig. 7b). Although chromatin interaction divergence among wheat species mainly occurred in one copy of homoeologous genes, there were 15 triad genes showing chromatin interaction divergence for all A, B, and D homoeologs among wheat species (Fig. 7a). Strikingly, more than half of these genes showed a positive correlation between chromatin interactions and expression levels (Fig. 7c). These results suggest chromatin interaction divergence occurred in different types of genes for different subgenomes during polyploidization and long-term evolution in wheat.

## Discussion

The interactions among *cis*-regulatory sequences such as enhancers and promoters mediate patterns of gene expression essential for plant development and adaption to various environments [36]. The hexaploid wheat (*Triticum aestivum*) has a large genome (16 Gb), only about 1% of which consists of protein-coding sequences [37]. It is important to identify functional regulatory elements in non-coding sequences and explore the impact of their spatial organization on gene expression in wheat. Although Hi-C and HiChIP are recently applied to study chromosome architecture in wheat [23, 24], a high-resolution interaction map among open chromatin regions is lacking and the role of chromatin interactions in gene regulation during wheat polyploidization is still poorly understood. In this study, we generated high-resolution open chromatin interaction maps for hexaploid wheat and its tetraploid and diploid relatives using OCEAN-C with low cost. The anchors of chromatin loops showed high chromatin accessibility and were concomitant with active histone modifications (Fig. 1f), confirming the enrichment of open chromatin regions in OCEAN-C experiments.

More chromatin interactions were observed in the D subgenome compared with the A and B subgenomes (Fig. 2b), which is consistent with higher chromatin accessibility and lower levels of repressive histone marks in the D subgenome [28, 38]. The hybridization between A and B genomes occurred 0.36 to 0.50 million years ago, but D genome merged with AB genomes about 10,000 years ago. Hybridization and polyploidization could induce significant epigenetic changes and reduce epigenetic divergence between subgenomes in plants [39, 40]. For example, the epigenetic difference of homoeologs is obviously smaller in tetraploid cottons than that in diploid cottons [39]. Long-time evolution may reduce epigenetic divergence between A and B subgenomes. The D subgenome may still largely maintain epigenetic status as diploid species after short-time evolution. The divergence of chromatin accessibility and chromatin interactions between D and AB subgenomes may be reduced during long-time evolution of hexaploid wheat. We found DNA transposons rather than retrotransposons were enriched in loop anchors (Fig. 1 g). This finding is consistent with that DNA transposons are prone to locate in the accessible regions [28]. The wheat genome has a rarely high proportion of DNA transposons compared with other grass genomes and the amplified DNA transposons are overrepresented in the chromosome distal regions [40, 41]. Strikingly, chromatin loops were predominately distributed in distal ends of chromosomes (Fig. 1e). These results suggest some DNA transposons in euchromatin have the potential to evolve into regulatory elements to regulate gene expression through chromatin loops.

Binding of transcription factors in regulatory elements could recruit RNA polymerase and the basal transcriptional machinery to regulate gene expression [42, 43]. We found binding motifs of various transcription factors were enriched in the distal HOCIs, which could interact with multiple anchors in wheat (Fig. 3a). The genes linked by TF-binding motif enriched HOCIs showed higher expression levels and lower coefficient of expression variance than the genes linked by other loops (Fig. 3b,c), which indicates that transcription factors may coordinate expression of many downstream genes through direct binding HOCIs. A previous study reveals that biosynthetic gene clusters are embedded in local hot spots of 3D contacts in *Arabidopsis thaliana* [44]. Indeed, distinct GO terms were enriched in genes linked by different chromatin loop networks (Additional file 2: Fig. S4a). The genes linked by the same HOCIs or chromatin loop networks may be involved in related metabolism processes.

Although divergence of transcription and chromatin interactions between homoeologous genes was observed in hexaploid wheat (Fig. 4b), we did not find a significant correlation between transcription divergence and chromatin interaction variations for homoeologs. On the one hand, the transcription divergence between homoeologs may be mainly mediated by variations of genetic sequences and epigenetic marks including DNA methylation and histone modifications. On the other hand, the difference of chromatin interaction in homoeologs was partly derived from chromatin structure variations (Fig. 4c–e). The chromatin interaction changes among homoeologous regions may be involved in maintaining genome stability but not gene regulation.

In previous studies [23, 24], the inter-subgenome interactions identified using multiple mapped reads were much more than those only using uniquely mapped reads. The interchromosomal interactions between homoeologous genomic regions may be due to mapping bias from multiple mapped reads in a previous report [24]. To exclude the effect of multiple mapping issues on the analysis of chromatin architecture, we only used uniquely mapped reads to identify chromatin loops. Indeed, rare interchromosomal loops (0.4%) linked homoeologous genes in this study. Therefore, multiple mapped reads should be excluded for analysis of chromatin interactions to reduce potential mapping errors.

Polyploidization induces rapid morphologic changes in wheat [45]. Changes of genetic sequences and epigenetic marks including DNA methylation and histone modifications are reported to be involved in gene regulation and phenotypic changes during wheat polyploidization [40, 45, 46]. By comparing hexaploid wheat with tetraploid and diploid relatives, we found a positive correlation between chromatin interaction changes and expression changes (Fig. 7), suggesting chromatin interactions are also involved in gene regulation during wheat polyploidization. Although chromatin interaction changes were widely observed in hexaploid wheat compared with tetraploid and diploid relatives, only limited triads showed chromatin interaction changes in all three homoeologs, which indicates chromatin interaction changes may contribute to functional differentiation of homoeologs after polyploidization by expression regulation. In summary, we explored the potential role of open chromatin interactions in gene expression and the effect of structure variations on spatial topology of open genomic regions during polyploidization and evolution of wheat. The findings and approaches described herein provide insightful clues for genome evolution of polyploid plants and epigenetic breeding of important crops.

## Conclusions

In summary, we investigate chromatin interaction changes between hexaploid wheat and its tetraploid and diploid relatives using OCEAN-C with low cost and high resolution. Our results show that the genomic structural variations contribute to chromatin interaction divergence among homoeologous genes. The chromatin topology changes mediate expression alteration of genes during polyploidization and evolution of wheat.

## Methods

### Plant materials and growth conditions

The bread wheat (*Triticum aestivum*) cultivar “Chinese Spring”, natural allotetraploid wheat (*T. turgidum L.* subsp. *durum*, AABB), and *Aegilops tauschii* subsp. *strangulata* (line RL5288, DD) were used in this study. The seeds were germinated in water for 3 days at 22 °C, then transferred to soil and grown under 18 °C/16 °C in day/night. Leaves in five-leaf stage were harvested and used for the construction of Hi-C, OCEAN-C, ChIP-seq, ATAC-seq, and RNA-seq libraries.

### OCEAN-C library construction

Hi-C libraries were constructed according to a published protocol [15]. About 0.5 g leaves for each replicate were harvested and immediately cross-linked in 1% formaldehyde buffer (10 mM potassium phosphate, 50 mM NaCl, 0.1 M sucrose, and 1% formaldehyde) for 30 min. Glycine buffer (10 mM potassium phosphate, 50 mM NaCl, 0.1 M sucrose, and 0.15 M glycine) was added to quench the reaction. Crosslinked leaves were rinsed thrice by deionized water and immediately frozen in liquid nitrogen. Leaves were ground into fine powder and transferred to nuclei isolation buffer (40% glycerol, 0.25 M sucrose, 20 mM HEPES, 1 mM MgCl_2_, 5 mM KCl, 0.25% TritonX-100, 0.1 mM PMSF, 1× Protease Inhibitor Cocktail (Roche) and 0.1% 2-mercaptoethanol). After mixing thoroughly, the slurry was kept on ice for 30 min and filtered by a 70-μm strainer. After centrifugation (3000×*g* for 5 min), the nuclei pellet was resuspended in 200 μl of 0.5% sodium dodecyl sulfate (SDS). Then the suspension was split into four tubes and incubated at 65 °C for 10 min. After adding 145 μl water and 25 μl 10% TritonX-100, each tube was incubated at 37 °C for 15 min to quench the SDS. The chromatin was digested by adding 25 μl 10× NEBuffer 3 and 50 U DpnII (NEB, R0543) at 37 °C overnight. On the following day, after incubating at 62 °C for 20 min to inactivate DpnII, the restriction fragments were filled by adding tagging buffer (10 μl 1 mM biotin-14-dCTP, 1 μl 10 mM dATP, 1 μl 10 mM dGTP, 1 μl 10 mM dTTP, 40 U Klenow (NEB, M0210) and 25 μl ddH_2_O) and incubated at 22 °C for 4 h. Next, filled fragments were proximally ligated by adding ligation buffer (663 μl water, 120 μl 10× T4 DNA ligase buffer, 100 μl 10% Triton X-100 and 2000 U T4 DNA Ligase (NEB, M0202)) and kept at 22 °C for 4 h. The mixture was centrifuged at 1000*g* for 3 min. The pellet was resuspended with 500 μl nuclei lysis buffer (10 mM Tris-HCl [pH 8.0], 2% Triton X-100, 1% SDS, 100 mM NaCl, and 1 mM EDTA) and was kept on ice for 30 min with occasional stirring. The mixture was sonicated to an average DNA fragment size of 300–400 bp (Instrument: Covaris LE220 (Covaris), Duty Cycle: 20, PIP: 50, Cycles/Burst: 200, Time: 100 s). Add 500 μl phenol-chloroform-isoamylalcohol (25:24:1, pH ≥ 7.8) and vortex for 30 s. After centrifugation at 13,000 g for 5 min at room temperature, the supernatants were extracted and purified by phenol-chloroform-isoamylalcohol (25:24:1, pH ≥ 7.8) again. The aqueous layer was transferred to a new 1.5-ml tube. RNase A (5 μl) was added following 30 min of incubation at 37 °C. Proteinase K (10 μl) was added and the tubes were kept at 55 °C for 1 h and then at 65 °C for 2 h. DNA was purified using QIAquick PCR Purification Kit (Qiagen, 28104) and then pulled down by Dynabeads MyOne Streptavidin T1 beads (Invitrogen, Cat No. 65601). Libraries were constructed using NEBNext Ultra II DNA Library Prep Kit (NEB, E7645L) and sequenced on NovaSeq platform (Illumina) for 150 bp paired-end reads.

### ChIP-seq library construction

The library construction of ChIP-seq with two biological replicates was performed as previous described [47]. Chromatin immunoprecipitation was performed using antibodies against H3K27ac (Abcam, ab4729) and H3K14ac (Abcam, ab52946). Library construction was performed using NEBNext® Ultra™ II DNA Library Prep Kit for Illumina (NEB, E7645L) according to the manufacturer’s instructions and sequenced on NovaSeq platform (Illumina) for 150 bp paired-end reads.

### ATAC-seq library construction

The ATAC-seq libraries were constructed according to the previous study with some modifications [48]. About 0.2 g leaves per replicate were collected and immediately chopped in 2 ml of pre-chilled lysis buffer (25 mM Tris-HCl pH=8, 0.44 M Sucrose, 10 mM MgCl_2_, 0.1% Triton X-100, 2 mM Spermine, 1 mM PMSF, 1× Cocktail, 10 mM 2-Me). The mixture was filtered by 40 μm strainer and washed by 1 ml nuclei extraction buffer 2 (25 mM Tris-HCl pH=8, 0.44 M Sucrose, 10 mM MgCl2, 0.1% Triton X-100, 1× cocktail (Roche), 10 mM 2-Me). Following resuspension by 300 μl nuclei extraction buffer 3 (1.7 M Sucrose, 10 mM Tris–HCl, 2 mM MgCl2, and 0.15% Triton X-100 and 1× Cocktail), the nuclei suspension was loaded on the surface of 600 μl nuclei extraction buffer 3 and centrifuged at 2400*g* at 4 °C for 20 min. The purified nuclei were resuspended by 1 ml nuclei extraction buffer 1. The small fraction of nuclei were stained with 4,6-diamidino-2-phenylindole and loaded into a hemocytometer to calculate nuclei density. About 50,000 nuclei were incubated with Tn5 transposase (Vazyme, TD501) at 37 °C for 30 min. DNA was recovered by MinElute PCR Purification Kit (Qiagen, 28004) and amplified for 11-13 cycles. In addition, genomic DNA was used for library construction as input control. Libraries were sequenced on NovaSeq platform (Illumina) for 150 bp paired-end reads.

### ChIP-seq and ATAC-seq data analysis

Sequencing reads of H3K4me3, H3K27me3, and H3K9ac of Chinese Spring were obtained from a previous study [2], which are available in NCBI GEO under accession number GSE121903. Sequencing reads were cleaned using NGSQC Toolkit (versition 2.3; 2 A -l 80 -s 20) and cutadapt (version 1.11). The reads of ChIP-seq and ATAC-seq from *Triticum aestivum* were mapped to the genome sequence of *Triticum aestivum cv.* Chinese Spring (IWGSC RefSeq v1.0) using bowtie2 (version 2.2.9) with default setting. The concordantly mapped reads (MAPQ> 10) were kept and PCR duplication was further removed with Picard. Correlation analysis between the two biological replicates of each mark was performed using deepTools2 (3.1.0.). Peaks of ChIP-seq libraries were detected using MACS2 (2.1.2) with the parameter “-f BAM --nomodel --bw 300 --SPMR -q 0.05”. The peaks of ATAC-seq were identified using MACS2 with the parameter “-q 0.01 -f BAM --nomodel --extsize 200 --shift 100”. The genomic DNA libraries were used as controls. The number of reads in each window was normalized against the total number of reads (RPM, Reads per Million Mapped Reads). Peak-associated genes were defined as genes with a peak within or near the gene body (±3 kb).

### RNA-seq data analysis

RNA-seq reads were mapped to genome sequence of *Triticum aestivum cv.* Chinese Spring (IWGSC RefSeq v1.0) using HISAT2 with default parameters. Only uniquely mapped reads were kept. RNA-seq reads were normalized to FPKM. The reads from two compared groups were normalized by their respective size factors, which were analyzed by DESeq package with the parameter “estimateSizeFactors.” Fold change (> 2) and *P* value < 0.05 were used to identify differentially expressed genes (DEGs).

### OCEAN-C data processing

OCEAN-C reads of *Triticum aestivum*, *T. durum*, and *Ae. tauschii* were mapped to the reference genome (Chinese Spring (IWGSC RefSeq v1.0) for *T. aestivum*, AB subgenomes of Chinese Spring (IWGSC RefSeq v1.0) for *T. durum*, D subgenome of Chinese Spring (IWGSC RefSeq v1.0) for *Ae. tauschii* ) using HiC-Pro (version 2.11.1) [49] with the parameter “MIN_MAPQ = 5”. Low quality mapped reads (mapping quality (MAPQ) < 5) and duplication were discarded. Self-circle, dangling-end, re-ligation, and dumped reads were removed. The remaining read pairs were used to call peaks by MACS2 (version 2.1.2) with the parameters “-f BAM --shift -75 --extsize 150 --nomodel -B --SPMR”. The re-sequencing reads of *T. aestivum*, *T. durum*, and *Ae. tauschii* were used as controls. The peaks of *T. aestivum* were used as an anchor to identify open chromatin interactions by hichipper [50]. To identify high-confidence chromatin interactions, we removed loops with genomic span < 10 kb. Chromatin interactions with at least three interacted read pairs and FDR < 0.05 were defined as high-confidence intrachromosomal and interchromosomal interactions. The anchors within 3 kb around genes were annotated as proximal peaks (P) and those anchors reside more than 3 kb away from genes were annotated as distal peaks (D). The loops were classified into P-P interaction (PPI), P-D interaction (PDI), and D-D interaction (DDI) accordingly. For any three PPIs, if anchors on one side of PPI loops were homoeologous genes between A, B, and D subgenomes and anchors on another side of PPI loops were also homoeologous genes, these PPIs were defined as conserved PPIs among all three subgenomes.

### Identification of triad genes with subgenomic bias

High-confidence gene models from the IWGSC (version 1.0) were used for defining triad genes. Homoeologous genes between each pair of A, B, and D subgenomes were identified as previously described [38]. The homoeolog groups with only one gene copy in each subgenome were defined as triads.

A previously described ternary plot-based method was applied for defining bias of histone modifications, ATAC-seq, gene expression, and interaction patterns in triads [38]. Euclidean distances of each gene along the three angles of the ternary plot were determined based on the fraction of the reads mapped to the given gene triad. All triads were divided into seven subgenome biased groups: a balanced group (BL), with similar modification or interaction level across the three homoeologs, and six dominant or suppressed groups with higher or lower levels in one homoeolog.

### Detection of TF-binding motifs

To detect enriched transcription factor-binding motifs in the proximal and distal regions, we first detected the proximal (3 kb upstream of the nearest TSS) and distal regions that interacted with gene loci. The regions interacting with more than 5 loci were considered as HOCIs. The motifs were then scanned against the proximal and distal regions using MEME (version 5.3.2) [51] software. The open chromatin regions were used as input. The motifs with *E* value less than 0.01 were defined as enriched motifs.

### ChIN analysis and visualization.

The igraph library in R software was used to construct chromatin network components (ChINs) (parameter: cluster_walktrap) and calculate the degree of each node (parameter: degree). The nodes and edges of each ChIN present the peaks and chromatin interactions. The Cytoscape [52] software was used to visualize ChINs.

### Identification of breakpoints of structural variation and PAVs

The genomes of bread wheat [37], *T. durum* [53] and *Ae. tauschii* [54], were compared using MUMmer (version 3, nucmer --mum -l 100 -c 1000-d 10) [55]. Then the results were filtered by delta-filter (-i 95 -o 95). To ensure the accuracy of breakpoints, only the structural variations longer than 1 Mb were retained.

Presence/absence variations (PAVs) were identified using the published methods [56]. We extracted unaligned regions between bread wheat and its tetraploid and diploid relatives from the “show-diff” command in MUMmer3 (version 3). These sequences were then filtered by discarding those overlapping with gap regions in the respective genome. The candidate PAV regions were retained by further removing regions of *T. aestivum* supported by resequencing data of *T. durum* and *Ae. tauschii.*

### Identification of differentially interacted loops between species

OCEAN-C peaks obtained from *T. aestivum*, *T. durum*, and *Ae. tauschii* were merged as a master peak list. The master peak list was used to call open chromatin loops using OCEAN-C reads by hichipper (--min-dist 10000) for each species [50]. The interaction intensity of each loop was normalized to counts per million mapped reads (CPM) for each biological replicate of each species. The differentially interacted intrachromosomal loops between species were identified using edgeR with FDR < 0.05 and fold-change > 1.5 [57].

### Identification of differentially interacted genes between species

The chromatin interaction intensity of each gene was normalized as counts per million mapped reads (CPM) for each biological replicate of *T. aestivum*, *T. durum*, and *Ae. tauschii.* The differentially interacted genes in *T. aestivum* compared with *T. durum* and *Ae. tauschii* were identified using edgeR with FDR < 0.05 and fold-change > 1.5 [57].

### 3D-FISH

3D DNA fluorescence in situ hybridization (3D-FISH) was performed according to previous study [58] with some minor modifications. 3D-FISH was performed using leaves of bread wheat at five-leaf stage. The tissue fixation and nuclei isolation was performed following instructions of the Hi-C method. After nuclei isolation, the pellet was resuspended with 300 μl of 1× PBS containing 0.5% triton X-100. The suspension was added to a Silanized slide (CITOGLAS, 188105 W) and kept at 4 °C for 20 min. The exons in two spatially proximate loci were used as probes labeled with different colors (Chr5A: 482–482.1 Mb: Fluorescein-12-dUTP, Thermo Fisher, R0101; Chr7B: 622.4–622.5 Mb: Texas Red-12-dUTP, Invitrogen, C7631). The 15-μl hybridization mixture (50% formamide, 2×SSC, 10% dextran sulfate, 0.3 μg/μl salmon sperm DNA, 2 μl probes) was denatured at 105 °C for 13 min, then kept at − 20 °C for 10 min. The slides were denatured by steeping in 70% ethanol with 0.15 mol/L NaOH for 5 min, and then in 70% ethanol for 10 min and 100% ethanol for 5 min. The 15 μl of denatured probe mixture was added to the denatured slide, which was then kept in a moist box at 37 °C overnight. After washing by ddH_2_O for 10 min, the slides were mounted in the Vectashield antifade solution (Vector, H-1200). The distance of signals was checked from 50 nuclei using a laser scanning confocal microscope Zeiss LSM780 with OLYMPUS cellSens Standard software.

## Supplementary Information

**Additional file 1: Supplementary tables S1-S3** and **S7**.

**Additional file 2: Supplementary Figure S1-S6**.

**Additional file 3: Table S4** The intrachromosomal open chromatin loops.

**Additional file 4: Table S5** The triads with chromatin interaction bias.

**Additional file 5: Table S6** The interchromosomal open chromatin loops.

**Additional file 6: Table S8** The differentially interacted intrachromosomal loops between bread wheat and *T. durum.*

**Additional file 7: Table S9** The differentially interacted intrachromosomal loops between bread wheat and *Ae. tauschii.*

**Additional file 8.** Review history.

## Data Availability

Sequencing data of Hi-C, OCEAN-C, ATAC-seq, RNA-seq, and ChIP-seq for H3K27ac and H3K14ac are available at Genome Sequence Archive in National Genomics Data Center https://bigd.big.ac.cn/gsa under accession number CRA003731 [59]. ChIP-seq for H3K27me3, H3K4me3, and H3K9ac using bread wheat leaf were generated by a previous study [2] and are available in NCBI GEO under accession number GSE121903.
