# Supplementary material for: Open chromatin interaction maps reveal functional regulatory elements and chromatin architecture variations during wheat evolution
Source: Genome Biol. 2022 Jan 24;23:34. doi: 10.1186/s13059-022-02611-3 (PMC8785527; doi:10.1186/s13059-022-02611-3)
Supplement: Supplementary file 1 — Additional file 1: Supplementary tables S1-S3 and S7. [file 13059_2022_2611_MOESM1_ESM.docx]

Table S1. Summary statistics of OCEAN-C libraries

|  | CS-1 | CS-2 | *T.durum*-1 | *T.durum*-2 | *Ae. tauschii*-1 | *Ae. tauschii*-2 |  |
| --- | --- | --- | --- | --- | --- | --- | --- |
| Total pairs | 470,323,205 | 414,817,748 | 424,933,298 | 491,773,319 | 198,534,210 | 209,640,461 |  |
| Unmapped | 9,483,270 | 11,712,727 | 25,135,489 | 30,063,179 | 9,652,061 | 13,826,278 |  |
| Low quality | 174,825,716 | 143,236,183 | 143,591,070 | 152,978,941 | 64,675,639 | 64,784,096 |  |
| Unique paired  % | 189,759,368  40.3 | 159,176,598  38.4 | 130,369,528  30.68 | 144,829,673  29.45 | 63,562,129  32.02 | 58,585,237  27.9 |  |
| Multiple pairs | 0 | 0 | 0 | 0 | 0 | 0 |  |
| Pairs with singleton | 96,254,851 | 100,692,240 | 125,837,211 | 162,130,748 | 60,644,381 | 72,444,850 |  |
| Low quality singleton | 0 | 0 | 0 | 0 | 0 | 0 |  |
| Valid interaction  % | 186,725,847  39.7 | 155,380,345  37.4 | 124,590,164  29.3 | 137,401,930  27.9 | 62,303,566  31.3 | 56,676,042  27 |  |
| Valid interaction pairs FF | 46,632,074 | 38,819,143 | 31,113,225 | 34,308,174 | 15,556,364 | 14,151,554 |  |
| Valid interaction pairs RR | 46,637,174 | 38,780,324 | 31,124,718 | 34,313,101 | 15,558,947 | 14,142,761 |  |
| Valid interaction pairs RF | 46,097,643 | 38,273,719 | 30,816,546 | 33,978,293 | 15,392,064 | 13,985,728 |  |
| Valid interaction pairs FR | 47,358,956 | 39,507,159 | 31,535,675 | 34,802,362 | 15,796,191 | 14,395,999 |  |
| Cis interaction  % | 123,117,869  26.2 | 108,702,268  26.2 | 63,260,349  14.9 | 65,801,588  13.3 | 36,540,892  18.4 | 38,853,783  18.5 |  |
| Cis shortRange (<20K)  % | 23,953,088  5.1 | 23,278,897  5.6 | 12,334,654  2.9 | 13,003,394  2.6 | 7,032,697  3.5 | 8,030,828  3.8 |  |
| Cis longRange (>20K)  % | 99,164,781  21.1 | 85,423,371  20.6 | 50,925,695  12 | 52,798,194  10.7 | 29,508,195  14.9 | 30,822,955  14.7 |  |
| Trans interaction  % | 24,470,226  5.2 | 19,904,703  4.8 | 13,960,346  3.3 | 14,678,250  3.0 | 6,519,758  3.3 | 6,711,089  3.2 |  |

Table S2. ChIP-seq and ATAC-seq used in this study

| Data type | Sample | Total reads | Aligned reads | % | MAPQ>=10 | Correlation |
| --- | --- | --- | --- | --- | --- | --- |
| ATAC-seq | CS leaf ATAC-seq rep1 | 161,457,418 | 153,061,632 | 94.8 | 100,036,180 | 0.96 |
| ATAC-seq | CS leaf ATAC-seq rep2 | 216,993,020 | 205,275,396 | 94.6 | 117,764,392 |  |
| Control of ATAC-seq | Rep1 | 279,455,169 | 273,866,065 | 98.0 | 169,261,658 | 0.99 |
| Control of ATAC-seq | Rep2 | 238,434,868 | 232,950,866 | 97.7 | 140,970,869 |  |
| ChIP-seq | CS leaf H3K27ac rep1 | 50,792,584 | 49,268,807 | 97.2 | 39,756,729 | 0.97 |
| ChIP-seq | CS leaf H3K27ac rep2 | 56,538,113 | 54,538,113 | 96.8 | 43,852,892 |  |
| ChIP-seq | CS leaf H3K14ac rep1 | 74,295,701 | 71,695,352 | 96.5 | 62,586,312 | 0.99 |
| ChIP-seq | CS leaf H3K14ac rep2 | 44,583,684 | 43,246,173 | 97.0 | 37,923,488 |  |
| ChIP-seq | CS leaf input rep1 | 47,826,503 | 46,056,923 | 96.3 | 35,673,019 | 0.95 |
| ChIP-seq | CS leaf input rep2 | 52,277,614 | 50,657,007 | 96.9 | 38,504,395 |  |

Table S3. Transcriptome data used in this study

| material |  | Total reads | Mapping reads | Unique mapping |
| --- | --- | --- | --- | --- |
| CS | Rep1 | 43,316,341 | 41,670,320 | 39,879,293 |
|  | Rep2 | 39,849,698 | 38,335,409 | 36,708,662 |
| *T. durum* | Rep1 | 37,064,083 | 35,915,096 | 29,488,823 |
|  | Rep2 | 40,621,602 | 39,727,926 | 25,381,289 |
| *Ae. tauschii* | Rep1 | 24,440,579 | 23,658,480 | 20794476 |
|  | Rep2 | 29,080,759 | 27,626,721 | 26,517,144 |

Table S7. Read statistics of OCEAN-C data of *Ae. tauschii*

| Reference |  | Total pairs | Unique paired | % | Valid interaction | % |
| --- | --- | --- | --- | --- | --- | --- |
| D subgenome of *T. aestivum* | Rep-1 | 198,534,210 | 63,562,129 | 32.02 | 62,303,566 | 31.3 |
|  | Rep-2 | 209,640,461 | 58,585,237 | 27.9 | 56,676,042 | 27 |
| *Ae. tauschii genome AL8* | Rep-1 | 198,534,210 | 57,967,792 | 29.2 | 56,809,010 | 28.6 |
|  | Rep-2 | 209,640,461 | 53,232,502 | 25.4 | 51,480,315 | 24.6 |
| *Ae. tauschii genome* AY17 | Rep-1 | 198,534,210 | 18,478,206 | 9.31 | 18,031,136 | 9.08 |
|  | Rep-2 | 209,640,461 | 17,501,101 | 8.34 | 16,808,273 | 8.02 |
| *Ae. tauschii genome* AY61 | Rep-1 | 198,534,210 | 53,730,883 | 27.06 | 52,624,857 | 26.5 |
|  | Rep-2 | 209,640,461 | 49,617,048 | 23.67 | 47,944,744 | 22.87 |
| *Ae. tauschii genome* T093 | Rep-1 | 198,534,210 | 19,339,141 | 9.74 | 18,880,145 | 9.51 |
|  | Rep-2 | 209,640,461 | 18,281,864 | 8.72 | 17,572,903 | 8.38 |
| *Ae. tauschii genome* XJ02 | Rep-1 | 198,534,210 | 18,558,740 | 9.35 | 18,110,360 | 9.12 |
|  | Rep-2 | 209,640,461 | 17,575,996 | 8.38 | 16,884,639 | 8.05 |
