# Supplementary material for: Open chromatin interaction maps reveal functional regulatory elements and chromatin architecture variations during wheat evolution
Source: Genome Biol. 2022 Jan 24;23:34. doi: 10.1186/s13059-022-02611-3 (PMC8785527; doi:10.1186/s13059-022-02611-3)
Supplement: Supplementary file 2 — Additional file 2: Supplementary Figure S1-S6. [file 13059_2022_2611_MOESM2_ESM.pptx]

## Slide 1
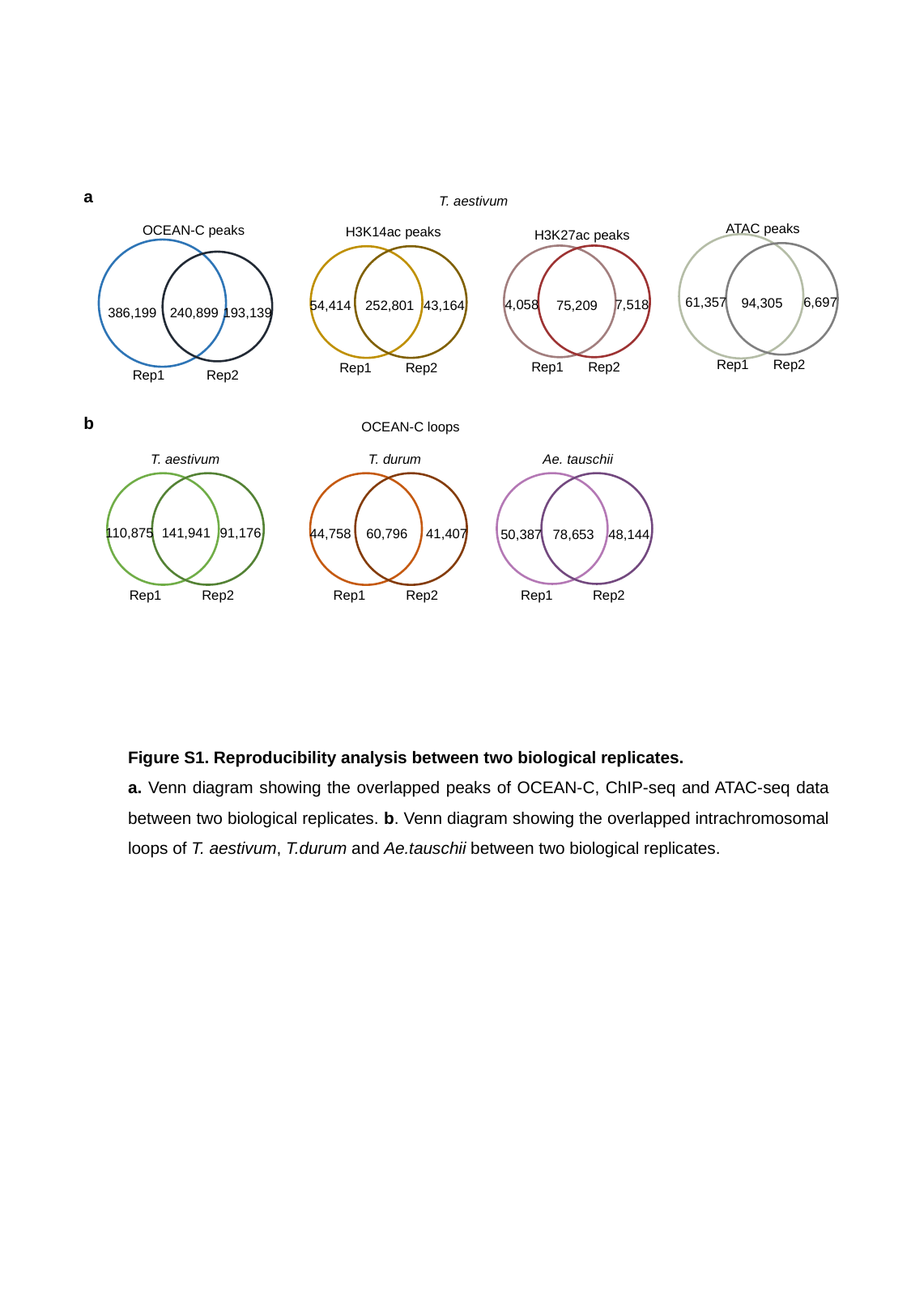

a
T. aestivum
ATAC peaks
OCEAN-C peaks
386,199
193,139
240,899
Rep1
Rep2
H3K14ac peaks
H3K27ac peaks
61,357
6,697
94,305
Rep1
Rep2
4,058
7,518
75,209
Rep1
Rep2
54,414
43,164
252,801
Rep1
Rep2
b
OCEAN-C loops
T. aestivum
T. durum
Ae. tauschii
110,875
91,176
141,941
Rep1
Rep2
44,758
41,407
60,796
Rep1
Rep2
50,387
78,653
48,144
Rep1
Rep2
Figure S1. Reproducibility analysis between two biological replicates.
a. Venn diagram showing the overlapped peaks of OCEAN-C, ChIP-seq and ATAC-seq data between two biological replicates. b. Venn diagram showing the overlapped intrachromosomal loops of T. aestivum, T.durum and Ae.tauschii between two biological replicates.

## Slide 2
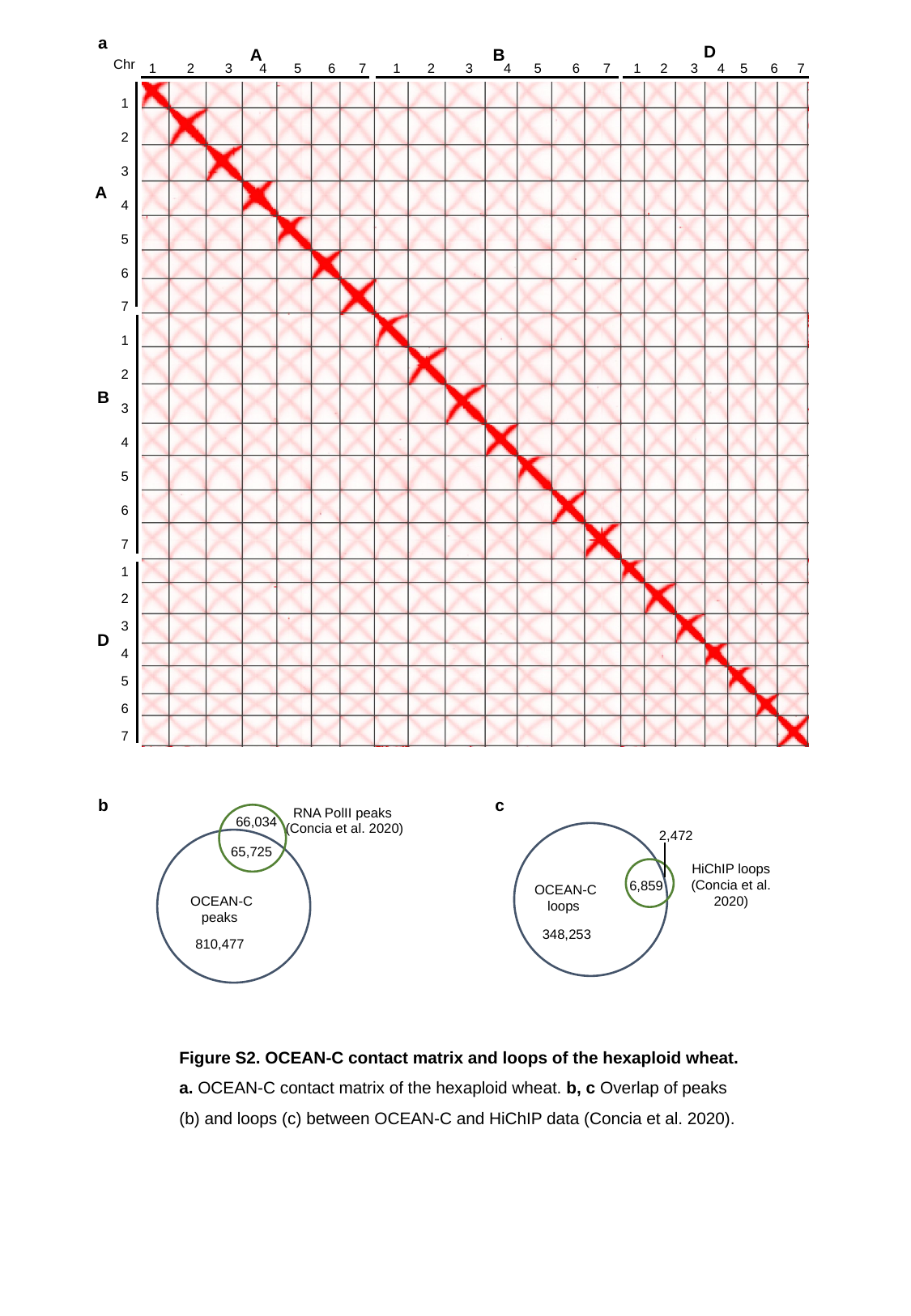

a
D
A
B
Chr
1 2 3 4 5 6 7 1 2 3 4 5 6 7 1 2 3 4 5 6 7
1
2
3
4
5
6
7
1
2
3
4
5
6
7
1
2
3
4
5
6
7
A
B
D
b
c
RNA PolII peaks
(Concia et al. 2020)
66,034
65,725
OCEAN-C peaks
810,477
2,472
HiChIP loops
(Concia et al. 2020)
6,859
OCEAN-C loops
348,253
Figure S2. OCEAN-C contact matrix and loops of the hexaploid wheat. a. OCEAN-C contact matrix of the hexaploid wheat. b, c Overlap of peaks (b) and loops (c) between OCEAN-C and HiChIP data (Concia et al. 2020).

## Slide 3
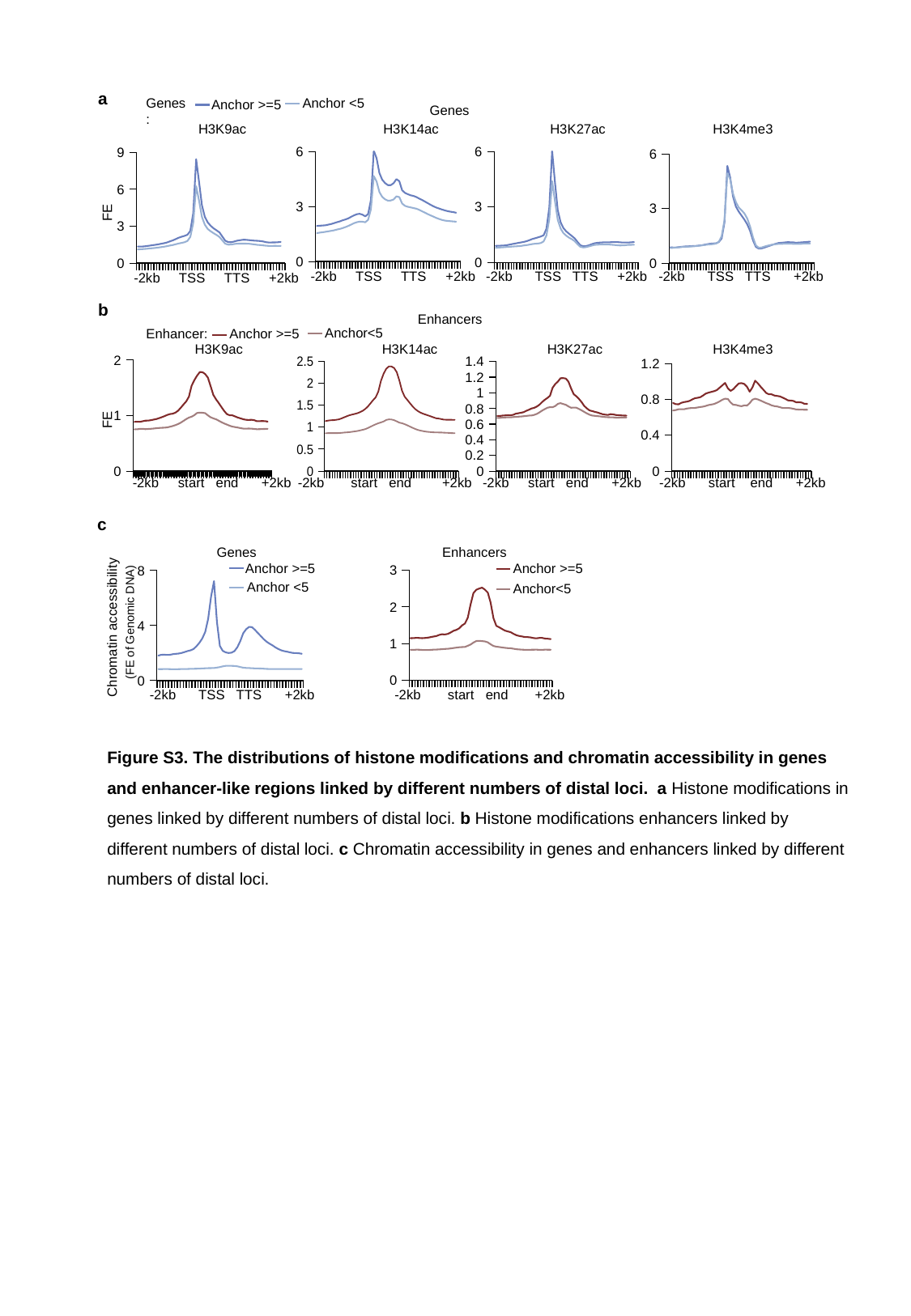

a
Genes:
Anchor <5
Anchor >=5
Genes
H3K9ac
H3K14ac
H3K27ac
H3K4me3
### Chart
| Category | High | Low |
|---|---|---|
### Chart
| Category | High | Low |
|---|---|---|
### Chart
| Category | High | Low |
|---|---|---|
### Chart
| Category | High | Low |
|---|---|---|FE
-2kb TSS TTS +2kb
-2kb TSS TTS +2kb
-2kb TSS TTS +2kb
-2kb TSS TTS +2kb
b
Enhancers
Anchor<5
Enhancer:
Anchor >=5
H3K9ac
H3K14ac
H3K4me3
H3K27ac
FE
### Chart
| Category | High | Low |
|---|---|---|
### Chart
| Category | High | Low |
|---|---|---|
### Chart
| Category | High | Low |
|---|---|---|
### Chart
| Category | High | Low |
|---|---|---|-2kb start end +2kb
-2kb start end +2kb
-2kb start end +2kb
-2kb start end +2kb
c
(FE of Genomic DNA)
Genes
Enhancers
Anchor >=5
Anchor >=5
### Chart
| Category | High_degree_Enhancer_CSATAC | Low_degree_Enhancer_CSATAC |
|---|---|---|
### Chart
| Category | High_degree_Gene_CSATAC | Low_degree_Enhancer_CSATAC |
|---|---|---|Anchor <5
Anchor<5
Chromatin accessibility
-2kb TSS TTS +2kb
-2kb start end +2kb
Figure S3. The distributions of histone modifications and chromatin accessibility in genes and enhancer-like regions linked by different numbers of distal loci. a Histone modifications in genes linked by different numbers of distal loci. b Histone modifications enhancers linked by different numbers of distal loci. c Chromatin accessibility in genes and enhancers linked by different numbers of distal loci.

## Slide 4
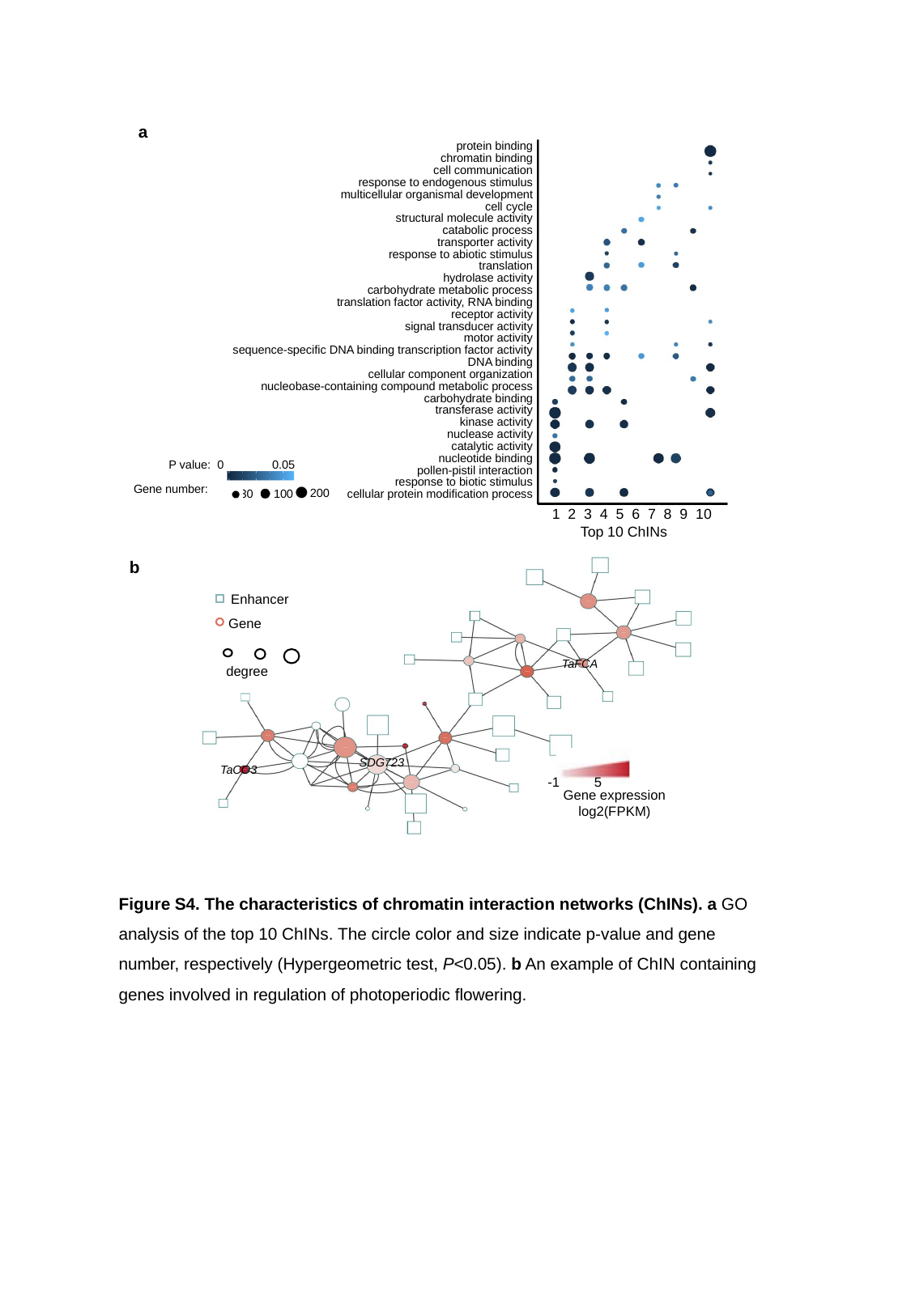

a
protein binding
chromatin binding
cell communication
response to endogenous stimulus
multicellular organismal development
cell cycle
structural molecule activity
catabolic process
transporter activity
response to abiotic stimulus
translation
hydrolase activity
carbohydrate metabolic process
translation factor activity, RNA binding
receptor activity
signal transducer activity
motor activity
sequence-specific DNA binding transcription factor activity
DNA binding
cellular component organization
nucleobase-containing compound metabolic process
carbohydrate binding
transferase activity
kinase activity
nuclease activity
catalytic activity
nucleotide binding
pollen-pistil interaction
response to biotic stimulus
cellular protein modification process
Gene number:
P value: 0 0.05
200
30
100
1 2 3 4 5 6 7 8 9 10
Top 10 ChINs
b
Enhancer
Gene
degree
TaFCA
-1 5
Gene expression
log2(FPKM)
SDG723
TaCO3
Figure S4. The characteristics of chromatin interaction networks (ChINs). a GO analysis of the top 10 ChINs. The circle color and size indicate p-value and gene number, respectively (Hypergeometric test, P<0.05). b An example of ChIN containing genes involved in regulation of photoperiodic flowering.

## Slide 5
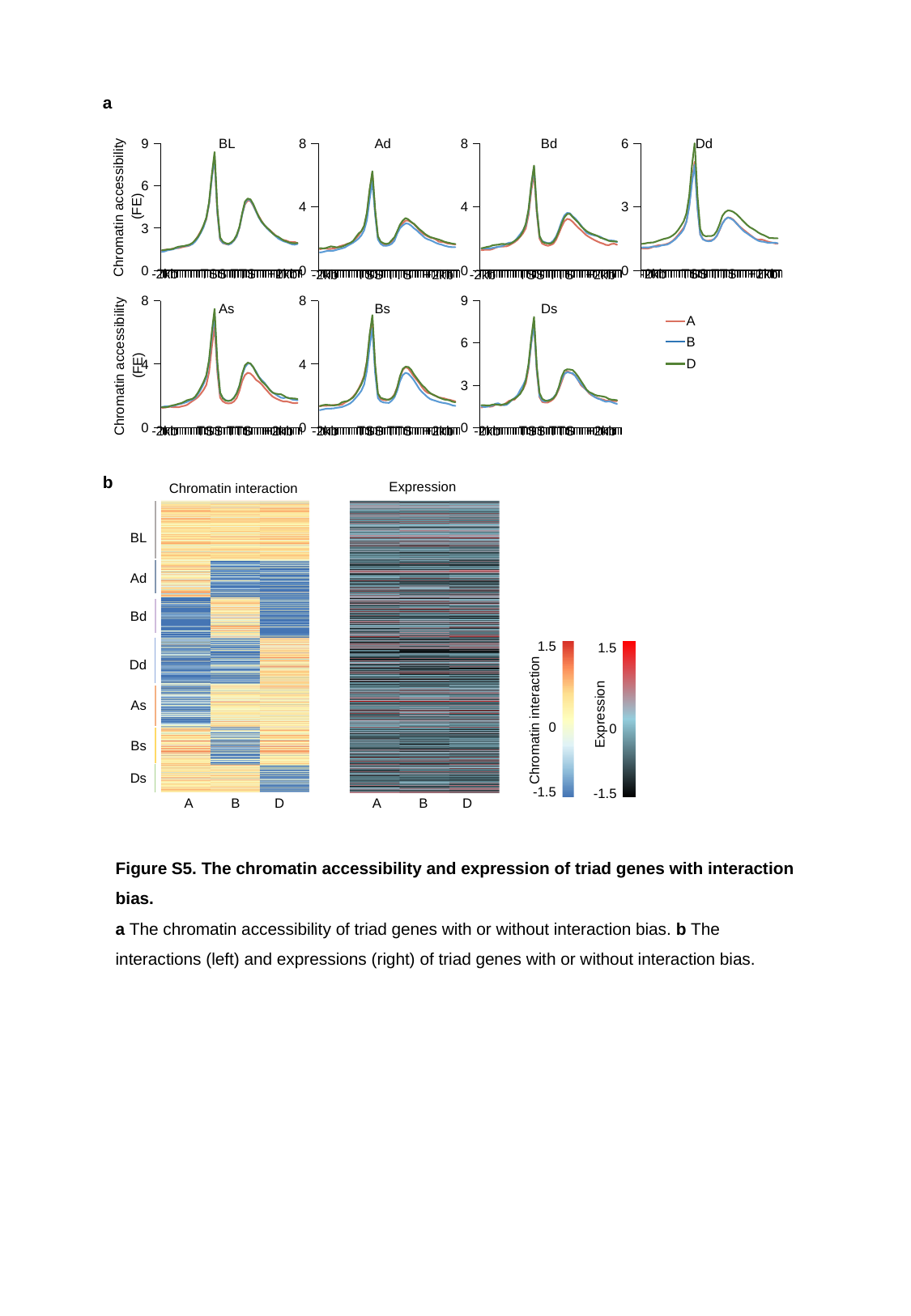

a
BL
Ad
Dd
Bd
### Chart
| Category | BLA | BLB | BLD |
|---|---|---|---|
### Chart
| Category | AdA | AdB | AdD |
|---|---|---|---|
### Chart
| Category | BdA | BdB | BdD |
|---|---|---|---|
### Chart
| Category | DdA | DdB | DdD |
|---|---|---|---|(FE)
Chromatin accessibility
-2kb TSS TTS +2kb
-2kb TSS TTS +2kb
-2kb TSS TTS +2kb
-2kb TSS TTS +2kb
### Chart
| Category | AsA | AsB | AsD |
|---|---|---|---|
### Chart
| Category | BsA | BsB | BsD |
|---|---|---|---|
### Chart
| Category | DsA | DsB | DsD |
|---|---|---|---|Ds
As
Bs
(FE)
A
B
Chromatin accessibility
D
-2kb TSS TTS +2kb
-2kb TSS TTS +2kb
-2kb TSS TTS +2kb
b
Expression
Chromatin interaction
BL
Ad
Bd
Dd
As
Bs
Ds
1.5
0
-1.5
1.5
0
-1.5
Expression
Chromatin interaction
A B D
A B D
Figure S5. The chromatin accessibility and expression of triad genes with interaction bias.
a The chromatin accessibility of triad genes with or without interaction bias. b The interactions (left) and expressions (right) of triad genes with or without interaction bias.

## Slide 6
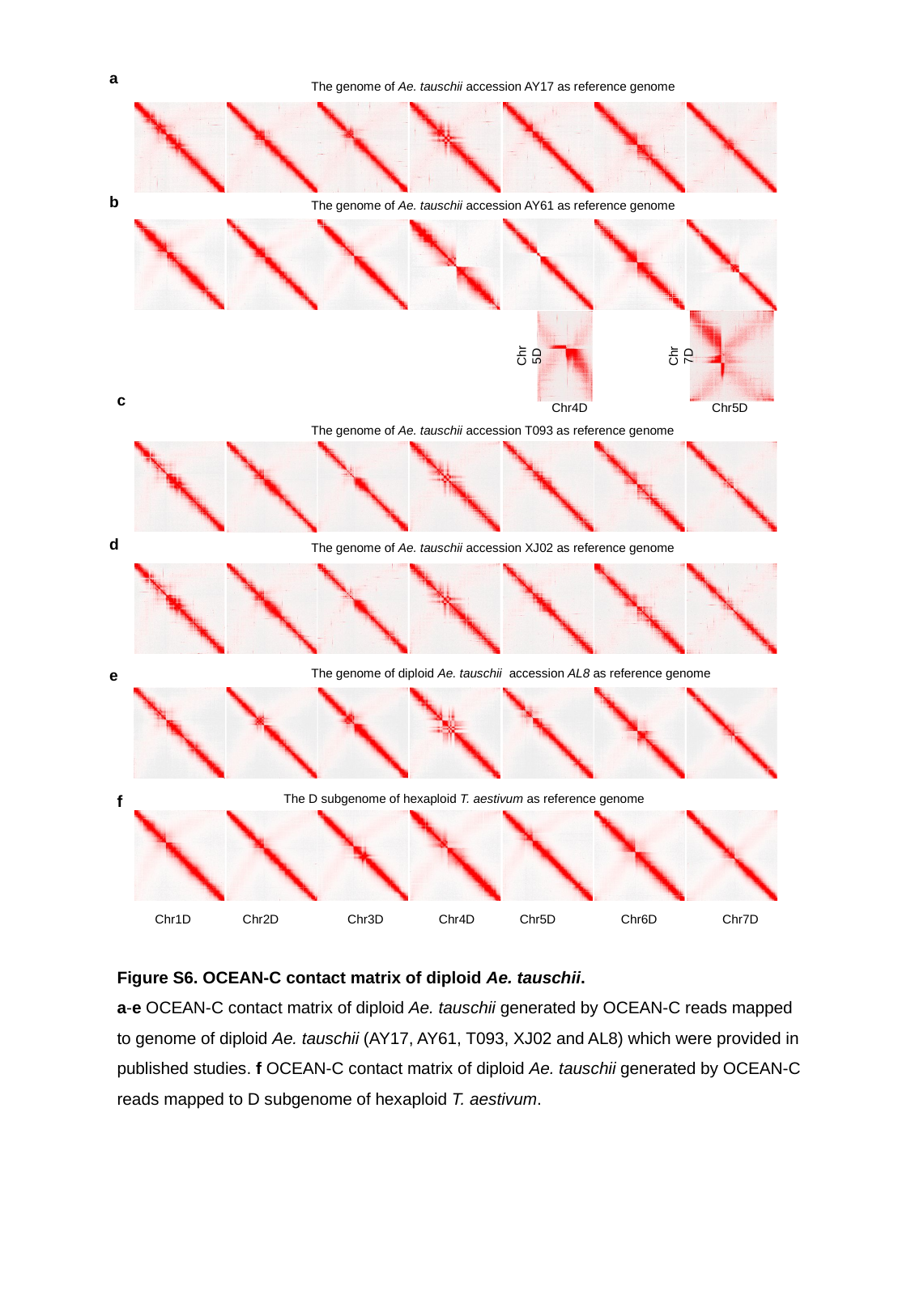

a
The genome of Ae. tauschii accession AY17 as reference genome
b
The genome of Ae. tauschii accession AY61 as reference genome
Chr5D
Chr7D
c
Chr4D
Chr5D
The genome of Ae. tauschii accession T093 as reference genome
d
The genome of Ae. tauschii accession XJ02 as reference genome
The genome of diploid Ae. tauschii accession AL8 as reference genome
The D subgenome of hexaploid T. aestivum as reference genome
e
f
 Chr1D Chr2D Chr3D Chr4D Chr5D Chr6D Chr7D
Figure S6. OCEAN-C contact matrix of diploid Ae. tauschii.
a-e OCEAN-C contact matrix of diploid Ae. tauschii generated by OCEAN-C reads mapped to genome of diploid Ae. tauschii (AY17, AY61, T093, XJ02 and AL8) which were provided in published studies. f OCEAN-C contact matrix of diploid Ae. tauschii generated by OCEAN-C reads mapped to D subgenome of hexaploid T. aestivum.
