# Supplementary material for: Open chromatin interaction maps reveal functional regulatory elements and chromatin architecture variations during wheat evolution
Source: Genome Biol. 2022 Jan 24;23:34. doi: 10.1186/s13059-022-02611-3 (PMC8785527; doi:10.1186/s13059-022-02611-3)
Supplement: Supplementary file 8 — Additional file 8. Review history. [file 13059_2022_2611_MOESM8_ESM.docx]

Review History

**First round of review**

**Reviewer 1**

**Are you able to assess all statistics in the manuscript, including the appropriateness of statistical tests used?**

No, I do not feel adequately qualified to assess the statistics.

**Comments to author:**

Yuan et al. developed OCEAN-C technique in wheat and used OCEAN-C data to explore the relationship between open chromatin interactions and gene transcription regulation. This study provides a resource for future functional genomics study in wheat, and also provides insights into chromatin architecture variations during polyploidization. I have some comments to improve this manuscript.

1. Fig. 1b: It seems that the authors want to show that OCEAN-C peaks were truly located in open chromatin regions, but I did not understand how this analysis was performed. Why not overlap OCEAN-C peaks with ATAC-Seq and ChIP-Seq peaks with active chromatin modifications?

2. How many OCEAN-C peaks and loops can be identified in both biological replicates? This information is very important for assessing the reproducibility of OCEAN-C experiment, since this technology has not been used in plants before.

3. It seems that the authors generated Hi-C data shown in the Methods, but this kind of data was not mentioned in the Results. I suggest the authors perform a reproducibility analysis of OCEAN-C loops relative to the Hi-C loops or HiChIP loops in previous study.

4. The authors should make a reproducibility analysis for the other raw sequencing data, especially for ATAC-Seq and ChIP-Seq.

5. Fig. 1d: Since these loops are associated with OCEAN-C peaks, which are enriched in the open chromatin regions as mentioned by the authors, PPI, PDI, and DDI should be enriched in the A compartment region. I am confused that how the authors perform the random analysis. Why the majority of random loops were included in the B compartment? It is not clear to me how the authors made this selection.

6. Fig. 1f: Were all the ChIP-Seq data generated in this study? Since only two antibodies (H3K27ac and H3K14ac) were used for experiment mentioned in the Methods.

7, Page 8, line 17: “The A, B and D subgenomes respectively contained 48,925, 45,904 and 59,564 PPIs”. The total number is 154,393. However, on page 7, line 60, it says: "The intrachromosomal loops contained 154,097 P-P interactions (PPI)". The authors should check this data.

8, Page 8, line 19, “2,464 PPIs were conserved among all three subgenomes”, how conserved PPIs were defined. The method should be specified.

9, Page 8, lines 32-35: “In wheat, the genes with loops, especially with both PPI and PDI, showed much higher expression levels than genes without loops.” I did not see the significance in Fig. 2d.

10, The section “Structural variations contribute to interaction divergence between subgenomes”: The authors suggested that homologous gene expression differences may be due to insertion/deletion or structural variation in intergenic regions. However, the authors mentioned a lot about the relationship between chromosomal loops and homologous gene expression differences, but did not find a direct link between insertion/deletion or structural variants and differences in gene expression. In addition, why the authors did not consider the impact of loops <=500Kb, since proximal regulation should be more important.

11. Page 10-11: “Previous wheat Hi-C data showed that interchromosomal interactions mainly occurred in homoeologous genomic regions [24]. However, we found rare interchromosomal loops (0.4%) linked homoeologous genes.” What is the possible reason for this difference?

12. Page 12. Fig. 7a: “For genes in triads, we respectively identified 481, 818 and 689 genes showing more chromatin interactions in A, B and D subgenomes of *T. aestivum* compared with *T. durum* and *Ae. tauschii* (Fig. 7a).” The method for identifying genes with different numbers of loops in diploid, tetraploid and hexaploid should be specified. Did the authors normalize the OCEAN-C data in the three species? In addition, the reproducibility analysis should be performed for diploid and tetraploid.

**Reviewer 2**

**Are you able to assess all statistics in the manuscript, including the appropriateness of statistical tests used?**

Yes, and I have assessed the statistics in my report.

**Comments to author:**

The authors use relatively novel open chromatin-conformation capture method OCEAN-C to identify global chromatin interactions in a hexaploid wheat. They analyze detected inter- and intra chromosomal loops and loop anchors in a detail, relating them to other chromatin features and gene expression. In addition, they attempt to compare the chromatin interactions between hexa- tetra- and diploid wheat species in order to explain gene expression divergence between homeologs during evolution.

The results are a valuable contribution to the field, providing high quality datasets that are of interest not only to crop research but generally to understanding the evolution of genome organization.

However, I have some corrections to the main text and Figures and objections to the Methods section. Also I have a few requests for re-analysis or providing more detail/statistics.

1.) Fig.1c: Fractions of PPI - swapped colors/legend PPI. Fig. 5a: The loops in the same subgenome were not visible on my screen/printout. Maybe remove them altogether?

2.) p. 1,line 40: Moreover, we found THAT structure....

3.) I am missing more details regarding the mapping of the OCEAN-C data. p.4, line 15: a total of 276M uniquely mapped reads does not correspond witht the S1 table (Unique paired 189,759,368 and 159,176,598). I understand that the HiC-Pro uses Bowtie2 for mapping, but the settings are unclear. The methods seciton mentions only MAPQ<5. My concern is possible involvement of satellite and transposable element repeat sequence involvement (besides the reported proximal TEs that may be quite diverged) that is likely in the large plant genomes. This may be neglected if multimapped reads are not treated accordingly. Have authors experimented with the settings (MAPQ>1; maybe "-best M 1 -strata" that can randomly distribute reads across repeats)?

What was the OCEAN-C resolution achieved?

4.) The method sections seem quite inconsistent in tools used for the same puropse and reporting the tool settings. For example, the old MACS version is used for ATAC and ChIP-seq data analysis without settings as compared to the newer version of MACS2 with the detalied settings for the OCEAN-C. Same with the duplicate removal - the old samtools version mentioned without any details, while only "duplication(s!) were discarded." for the OCEAN-C.

5.) Regarding the RNAseq data analysis, I am surprised to see the authors using the old, deprecated TopHat tools for mapping. Even the tool authors recommend using HiSat2 https://ccb.jhu.edu/software/tophat/index.shtml, or even better (reference-free) methods are avaliable nowadays. Publications serve as a template for some other scientists for doing their analysis, and I don`t think it is good to encourage using outdated algorithms even if the final result may not be affected seriously.

6.) Regarding the use of T. durum instead of the poorly assembled taushii: were the mapping stats so bad that the result would be unusable? What about correcting misassemblies in the Aegilops taushii by running 3D-DNA first instead of compromising by using T. durum that may bear some true rearrangements? Alterantively, there is a recent work by Zhou et al., 2021 who provided high-quality references which could be also used.

7.) With the FISH experiments, please provide some more detailed analysis - numbers of nuclei analyzed and distance values measured. One figure (5f) is not enough to support the inter-chromosomal loops. - STATISTICS required

8.) The peaks within 3 kb around genes were annotated as proximal peaks, but how many overlap with the TSSes? This is a very important piece of information. Especially for the motif search, I suggest separating those to reveal possible third category of "core promoter" interactions as distinct from "proximal enhancer" (if the resolution allows for that). Should TATA-box or other promoter motifs appear too in case the core promoters interact?

**Authors Response**

**Point-by-point responses to the reviewers’ comments:**

Reviewer #1: Yuan et al. developed OCEAN-C technique in wheat and used OCEAN-C data to explore the relationship between open chromatin interactions and gene transcription regulation. This study provides a resource for future functional genomics study in wheat, and also provides insights into chromatin architecture variations during polyploidization. I have some comments to improve this manuscript.

*Response: We appreciate the encouraging and constructive comments from this expert reviewer and have addressed the concerns in this revision. 1. Fig. 1b: It seems that the authors want to show that OCEAN-C peaks were truly located in open chromatin regions, but I did not understand how this analysis was performed. Why not overlap OCEAN-C peaks with ATAC-Seq and ChIP-Seq peaks with active chromatin modifications? Response: Thanks for this suggestion. We identified 891,128 OCEAN-C peaks and 179,762 ATAC-seq peaks in this study (Fig. 1b). As suggested, we examined the overlap between OCEAN-C peaks and ATAC-seq peaks in revision (Fig. 1b). More than half (53%) of ATAC-seq peaks were overlapped with OCEAN-C peaks, suggesting OCEAN-C peaks were mainly located in open chromatin regions.*

*For the overlap between OCEAN-C peaks and ChIP-seq peaks with active chromatin modifications, we showed these related data in Fig. 1f in initial submission. About 29- 44% of OCEAN-C peaks were overlapped with ChIP-seq peaks with active chromatin modifications.*

2. How many OCEAN-C peaks and loops can be identified in both biological replicates? This information is very important for assessing the reproducibility of OCEAN-C experiment, since this technology has not been used in plants before.

*Response: We agree with reviewer’s comment. There was high reproducibility between two replicates (Pearson correlation = 0.97). We identified 627,098 and 434,033 OCEAN-C peaks from each biological replicate, respectively. About 56% of peaks were observed in both replicates (Additional file 2: Figure S1a). In addition to OCEAN-C peaks, 252,816 and 233,117 intrachromosomal loops were identified in each biological replicate, respectively. About 61% of loops were overlapped between two biological replicates (Additional file 2: Figure S1b). These results suggest high reproducibility in OCEAN-C experiments. We have added related figures in Fig. S1 in revision.*

3. It seems that the authors generated Hi-C data shown in the Methods, but this kind of data was not mentioned in the Results. I suggest the authors perform a reproducibility analysis of OCEAN-C loops relative to the Hi-C loops or HiChIP loops in previous study.

*Response: This is a valid comment. When we started this project, 3D genome of wheat has not been published. We planned to use Hi-C data to confirm whether OCEAN-C data could represent high-order organization of wheat genome as Hi-C. As recent researches have revealed wheat chromosome architecture and found Rabl chromosome configuration in wheat genome [1], we didn’t use our Hi-C data to show wheat chromosome architecture in this study. In addition, low sequencing depth was obtained for Hi-C in this study. It’s not appropriate to compare OCEAN-C loops and Hi-C loops due to low resolution (40 kb) of Hi-C data. We decided to remove Hi-C data in revision. As suggested, we performed a reproducibility analysis of OCEAN-C loops using published RNA Pol II HiChIP data [1]. Using the same read mapping and loop calling strategies of OCEAN-C analysis, we re-analyzed RNA Pol II HiChIP data. The peaks of RNA Pol II ChIP-seq were used as anchor to identify open chromatin interactions by hichipper [2]. Chromatin interactions supported by at least three read pairs and FDR < 0.05 were defined as high-confidence intrachromosomal loops for RNA Pol II HiChIP data. Finally, 50% of RNA Pol II peaks were overlapped with OCEAN-C peaks (Additional file 2: Figure S2b). And 73.5% of HiChIP loops were overlapped with OCEAN-C loops (Additional file 2: Figure S2c). These results suggest OCEAN-C loops could capture most of RNA Pol II HiChIP loops. These results were updated in revision.*

4. The authors should make a reproducibility analysis for the other raw sequencing data, especially for ATAC-Seq and ChIP-Seq.

*Response: As suggested, we made a reproducibility analysis for the ChIP-Seq and ATAC-seq. There was high reproducibility between biological replicates (Pearson correlation = 0.95~0.99) for ATAC-seq, ChIP-seq and RNA-seq (Additional file 1: Table S2), and 56%~95% peaks were overlapped between two biological replicates (Additional file 2: Figure S1a). We have added corresponding results in revision.*

5. Fig. 1d: Since these loops are associated with OCEAN-C peaks, which are enriched in the open chromatin regions as mentioned by the authors, PPI, PDI, and DDI should be enriched in the A compartment region. I am confused that how the authors perform the random analysis. Why the majority of random loops were included in the B compartment? It is not clear to me how the authors made this selection.

*Response: The average length of OCEAN-C anchors was 4.6 kb. In initial submission, the windows at 5 kb were randomly selected using BEDTools from each chromosome. A total of 356,884 loops were randomly generated between these windows. However, we think random loops may be not appropriate for this analysis. To make it more clear, we examined compartment distribution of OCEAN-C anchors instead of OCEAN-C loops in revision. The windows at 5 kb were randomly selected as random anchors using BEDTools from each chromosome. The OCEAN-C anchors were mainly enriched in A compartments and random anchors were mainly distributed in B compartment (Fig. 1d). The method of random selection was added in figure legend.*

6. Fig. 1f: Were all the ChIP-Seq data generated in this study? Since only two antibodies (H3K27ac and H3K14ac) were used for experiment mentioned in the Methods.

*Response: ChIP-seq of H3K27ac and H3K14ac was generated in this study. ChIP-seq data of H3K4me3, H3K27me3 and H3K9ac in same T. aestivum accession Chinese Spring were obtained from previous study [3], which are available in NCBI GEO under accession number GSE121903. We have added corresponding explanation in Methods in revision.*

7, Page 8, line 17: “The A, B and D subgenomes respectively contained 48,925, 45,904 and 59,564 PPIs”. The total number is 154,393. However, on page 7, line 60, it says: "The intrachromosomal loops contained 154,097 P-P interactions (PPI)". The authors should check this data.

*Response: We apologize for this mistake. After checking, we revised this in revision as “The intrachromosomal loops contained 154,393 (43.2%) P-P interactions (PPI), 152,358 (42.7%) P-D interactions (PDI) and 50,133 (14.0%) D-D interactions (DDI)”.*

8, Page 8, line 19, “2,464 PPIs were conserved among all three subgenomes”, how conserved PPIs were defined. The method should be specified.

*Response: For any three PPIs, if anchors on one side of PPI loops were homoeologous genes between A, B and D subgenomes and anchors on another side of PPI loops were also homoeologous genes, these PPIs were defined as conserved PPIs among all three subgenomes. We have added corresponding explanation in Methods in revision.*

9, Page 8, lines 32-35: “In wheat, the genes with loops, especially with both PPI and PDI, showed much higher expression levels than genes without loops.” I did not see the significance in Fig. 2d.

*Response: To make it clear, we added the statistical analysis in revision as “In wheat, the genes with loops, especially with both PPI and PDI, showed much higher expression levels than genes without loops (Wilcoxon rank-sum test, P < 0.01) (Fig. 2d)” and added significant indication (**) in Fig. 2d.*

10, The section “Structural variations contribute to interaction divergence between subgenomes”: The authors suggested that homologous gene expression differences may be due to insertion/deletion or structural variation in intergenic regions. However, the authors mentioned a lot about the relationship between chromosomal loops and homologous gene expression differences, but did not find a direct link between insertion/deletion or structural variants and differences in gene expression. In addition, why the authors did not consider the impact of loops <=500Kb, since proximal regulation should be more important.

*Response: We apologize for this confusion. Actually, we didn’t find any correlation between expression bias of homoeologous genes and chromatin interaction divergence among subgenomes. In initial submission, we said “gene expression bias was also not observed in homoeologous genes with chromatin interaction divergence (Additional file 2: Figure S5b)”. We also discussed potential reasons in Discussion in initial submission as “Although divergence of transcription and chromatin interactions between homoeologous genes was observed in hexaploid wheat (Fig. 4b), we did not find significant correlation between transcription divergence and chromatin interaction variations for homoeologs. On the one hand, the transcription divergence between homoeologs may be mainly mediated by variations of genetic sequences and epigenetic marks including DNA methylation and histone modifications. On the other hand, the difference of chromatin interaction in homoeologs was partly derived from chromatin structure variations (Fig. 4c-e). The chromatin interaction changes among homoeologous regions may be involved in maintaining genome stability but not gene regulation”. In this section, we mainly focused on the effect of large genomic structural variations on chromatin interaction divergence not on gene expression bias among subgenomes. Homoeologous genes with balanced chromatin interactions showed similar fraction of long distance loop (>500kb) among subgenomes (Fig. 4c). Whereas genes with dominant chromatin interactions showed higher fraction of long distance loop (Fig. 4c). So we think sequence insertion/deletion or structural variation may induce generation of long distance loop (>500kb) to influence interaction divergence among subgenomes.*

11. Page 10-11: “Previous wheat Hi-C data showed that interchromosomal interactions mainly occurred in homoeologous genomic regions [24]. However, we found rare interchromosomal loops (0.4%) linked homoeologous genes.” What is the possible reason for this difference?

*Response: We explained possible reason of this difference in Discussion in revision as “In previous study [23,24], the inter-subgenome interactions identified using multiple mapped reads were much more than those only using uniquely mapped reads. The interchromosomal interactions between homoeologous genomic regions may be due to mapping bias from multiple mapped reads in previous report [23]. To exclude effect of multiple mapping issue on analysis of chromatin architecture, we only used uniquely mapped reads to identify chromatin loops. Indeed, rare interchromosomal loops (0.4%) linked homoeologous genes in this study. Therefore, multiple mapped reads should be excluded for analysis of chromatin interactions to reduce potential mapping errors”.*

12. Page 12. Fig. 7a: “For genes in triads, we respectively identified 481, 818 and 689 genes showing more chromatin interactions in A, B and D subgenomes of T. aestivum compared with T. durum and Ae. tauschii (Fig. 7a).” The method for identifying genes with different numbers of loops in diploid, tetraploid and hexaploid should be specified. Did the authors normalize the OCEAN-C data in the three species? In addition, the reproducibility analysis should be performed for diploid and tetraploid.

*Response: We added the related method in Methods in revision as “The chromatin interaction intensity of each gene was normalized as counts per million mapped reads (CPM) for each biological replicate of T. aestivum, T. durum and Ae. tauschii. The differentially interacted genes in T. aestivum compared with T. durum and Ae. tauschii were identified using edgeR with FDR < 0.05 and fold-change > 1.5”. As suggested, we performed reproducibility analysis of diploid and tetraploid OCEANC data. The pearson’s correlation coeffieient of OCEAN-C data between two biological replicates of Ae. tauschii and T. durum were 0.99 and 0.98, respectively. About 58%~60% of loops were overlapped between two replications (Additional file 2: Figure S1b). These results suggest high reproducibility of the biological replicates for Ae. tauschii and T. durum. We added related results in revision.*

Reviewer #2:

The authors use relatively novel open chromatin-conformation capture method OCEAN-C to identify global chromatin interactions in a hexaploid wheat. They analyze detected inter- and intra chromosomal loops and loop anchors in a detail, relating them to other chromatin features and gene expression. In addition, they attempt to compare the chromatin interactions between hexa- tetra- and diploid wheat species in order to explain gene expression divergence between homeologs during evolution. The results are a valuable contribution to the field, providing high quality datasets that are of interest not only to crop research but generally to understanding the evolution of genome organization. However, I have some corrections to the main text and Figures and objections to the Methods section. Also I have a few requests for re-analysis or providing more detail/statistics.

*Response: We appreciate the positive and encouraging and constructive comments from this expert reviewer and have addressed the concerns as follows.*

1.) Fig.1c: Fractions of PPI - swapped colors/legend PPI. Fig. 5a: The loops in the same subgenome were not visible on my screen/printout. Maybe remove them altogether?

*Response: Sorry for these mistakes. We revised the legend of Fig. 1c as “Fractions of PPI (green), PDI (orange) and DDI (purple) loops in all identified OCEAN-C loops” and removed loops between same subgenome in Fig. 5a.*

2.) p. 1,line 40: Moreover, we found THAT structure....

*Response: We revised it as “Moreover, we found that structure variations contributed to chromatin interaction divergence of homoeologs and chromatin topology changes between different wheat species.”*

3.) I am missing more details regarding the mapping of the OCEAN-C data. p.4, line 15: a total of 276M uniquely mapped reads does not correspond with the S1 table (Unique paired 189,759,368 and 159,176,598). I understand that the HiC-Pro uses Bowtie2 for mapping, but the settings are unclear. The methods seciton mentions only MAPQ<5. My concern is possible involvement of satellite and transposable element repeat sequence involvement (besides the reported proximal TEs that may be quite diverged) that is likely in the large plant genomes. This may be neglected if multimapped reads are not treated accordingly. Have authors experimented with the settings (MAPQ>1; maybe "-best M 1 -strata" that can randomly distribute reads across repeats)? What was the OCEAN-C resolution achieved? *Response: For p.4 line 15, it should be 276 million valid interaction pairs in Table S1. Sorry for this mistake. We revised this sentence as “A total of 276 million valid interaction pairs were obtained”.*

*The OCEAN-C reads were processed using HiC-Pro (version 2.11.1) with the parameter “MIN_MAPQ = 5”, which could remove mapped reads with low quality (MAPQ < 5). Meanwhile, HiC-Pro removes multiple mapped reads and duplicated reads by default. We added the detail of HiC-Pro parameters in revision. The parameter “-best M 1 - strata” belongs to software bowtie (version 1), which reports best one hit in multiple alignments. However, HiC-Pro uses bowtie2 for mapping. Bowtie2 reports one best hit in multiple alignments by default. As suggested, we re-mapped OCEAN-C reads using HiC-Pro with the parameter “MIN_MAPQ = 1”. We could only get 1.1% more valid interaction pairs using the parameter “MIN_MAPQ = 1” compared with the parameter “MIN_MAPQ = 5” (Table R1). The interaction matrix of OCEAN-C data using parameter “MIN_MAPQ = 1” or “MIN_MAPQ = 5” was similar to the Hi-C interaction matrix that was generated using uniquely mapped reads in previous study (Figure R1) [4]. These results indicate our OCEAN-C analysis have excluded multiple mapped reads.*

4.) The method sections seem quite inconsistent in tools used for the same puropse and reporting the tool settings. For example, the old MACS version is used for ATAC and ChIP-seq data analysis without settings as compared to the newer version of MACS2 with the detalied settings for the OCEAN-C. Same with the duplicate removal - the old samtools version mentioned without any details, while only "duplication(s!) were discarded." for the OCEAN-C.

*Response: As suggested, we re-analyzed the ATAC-seq and ChIP-seq data using MACS2 (2.1.2). The method was revised as “The reads of ChIP-seq and ATAC-seq from Triticum aestivum were mapped to the genome sequence of Triticum aestivum cv. Chinese Spring (IWGSC RefSeq v1.0) using bowtie2 (version 2.2.9) with default setting. The concordantly mapped reads (MAPQ > 10) were kept and PCR duplication was further removed with Picard. Peaks of ChIP-seq libraries were detected using MACS2 (2.1.2) with the parameter “-f BAM --nomodel --bw 300 -- SPMR -q 0.05”.*

*The peaks of ATAC-seq were identified using MACS2 with the parameter “-q 0.01 -f BAM --nomodel --extsize 200 --shift 100”. The genomic DNA libraries were used as controls”. Fig.1e-f, Fig.2c, Fig.4b, Additional file 2: Fig. S3, Fig. S5 and Additional file 1: Table S2 were updated in revision and all results are consistent with initial submission.*

5.) Regarding the RNAseq data analysis, I am surprised to see the authors using the old, deprecated TopHat tools for mapping. Even the tool authors recommend using HiSat2 https://ccb.jhu.edu/software/tophat/index.shtml, or even better (reference-free) methods are avaliable nowadays. Publications serve as a template for some other scientists for doing their analysis, and I don`t think it is good to encourage using outdated algorithms even if the final result may not be affected seriously.

*Response: As suggested, we re-analyzed the RNA-seq data using HISAT2 and the method was revised as “RNA-seq reads were mapped to genome sequence of Triticum aestivum cv. Chinese Spring (IWGSC RefSeq v1.0) using HISAT2 with default parameters. Only uniquely mapped reads were kept. RNA-seq reads were normalized to FPKM. The reads from two compared groups were normalized by their respective size factors, which were analyzed by DESeq package with the parameter “estimateSizeFactors”. Fold change (> 2) and P value < 0.05 were used to identify differentially expressed genes (DEGs)”. Fig. 2d-e, Fig. 3b-c, Fig.4b, Fig.5d, Fig. 6, Fig7, Additional file 2: Fig. S5 and Additional file 1: Table S3 were updated in revision.*

6.) Regarding the use of T. durum instead of the poorly assembled taushii: were the mapping stats so bad that the result would be unusable? What about correcting misassemblies in the Aegilops taushii by running 3D-DNA first instead of compromising by using T. durum that may bear some true rearrangements? Alterantively, there is a recent work by Zhou et al., 2021 who provided high-quality references which could be also used.

*Response: The genome of T. durum is AABB. We mapped OCEAN-C data of Ae. tauschii to D subgenomes of T. aestivum (AABBDD) rather than T. durum genome.*

*As suggested, we mapped OCEAN-C reads of Ae. taushii to different genomes including the references assembled by Zhou et al. 2021 (AY17, AY61, T093 and XJ02) (Table R2) [5]. When D subgenome of hexaploid T. aestivum as reference, the number of uniquely mapped reads was highest (Table R2) and anomalous chromatin interaction structures in matrix was least (Figure R2). So we still decided to use subgenomes (AABB and DD) of T. aestivum as reference genomes to map OCEAN-C data of the T. durum and Ae. tauschii.*

7.) With the FISH experiments, please provide some more detailed analysis - numbers of nuclei analyzed and distance values measured. One figure (5f) is not enough to support the inter-chromosomal loops. - STATISTICS required.

*Response: We analyzed 50 nuclei of five-leaf stage leaves using Confocal Microscopy. The signals of two loci (Chr5A: 482.0-482.1 Mb; Chr7B: 622.4-622.5 Mb) were observed on the same focal plane in 84% nuclei. The average distance of these two loci were 0.55 μm, which indicates these two loci were spatially proximate. We added measured distances of these two loci in Fig. 5g and indicate analyzed nuclei number in figure legend in revision.*

8.) The peaks within 3 kb around genes were annotated as proximal peaks, but how many overlap with the TSSes? This is a very important piece of information. Especially for the motif search, I suggest separating those to reveal possible third category of "core promoter" interactions as distinct from "proximal enhancer" (if the resolution allows for that). Should TATA-box or other promoter motifs appear too in case the core promoters interact?

*Response: These are good suggestions. Most proximal hubs of open chromatin interactions (56%) were overlapped with TSSes. However, in our study, the average length of anchors was 4.6 kb. Only 21 proximal hubs of open chromatin interactions were totally included in TSS regions (1 kb upstream and 200 bp downstream of transcription initiation site). Most proximal hubs of open chromatin interactions were overlapped with both core promoter (1 kb upstream and 200 bp downstream of TSS) and proximal promoter (-3 kb ~ -2 kb of TSS). So we cannot separate hubs of open chromatin interactions or identify potential motifs in core promoters.*

*References 1. Concia L, Veluchamy A, Ramirez-Prado JS, Martin-Ramirez A, Huang Y, Perez M, Domenichini S, Rodriguez Granados NY, Kim S, Blein T, et al: Wheat chromatin architecture is organized in genome territories and transcription factories. Genome Biol 2020, 21:104.*

*2. Lareau CA, Aryee MJ: hichipper: a preprocessing pipeline for calling DNA loops from HiChIP data. Nat Methods 2018, 15:155-156.*

*3. Li Z, Wang M, Lin K, Xie Y, Guo J, Ye L, Zhuang Y, Teng W, Ran X, Tong Y, et al: The bread wheat epigenomic map reveals distinct chromatin architectural and evolutionary features of functional genetic elements. Genome Biol 2019, 20:139.*

*4. Jia J, Xie Y, Cheng J, Kong C, Wang M, Gao L, Zhao F, Guo J, Wang K, Li G, et al: Homologymediated inter-chromosomal interactions in hexaploid wheat lead to specific subgenome territories following polyploidization and introgression. Genome Biol 2021, 22:26.*

*5. Zhou Y, Bai S, Li H, Sun G, Zhang D, Ma F, Zhao X, Nie F, Li J, Chen L, et al: Introgressing the Aegilops tauschii genome into wheat as a basis for cereal improvement. Nat Plants 2021, 7:774-786.*

**Second round of review**

**Reviewer 1**

The authors have addressed all my concerns, and I have no other suggestions. The manuscript is largely improved.

**Reviewer 2**

The only point that was not responded to my satisfaction is #6.) Regarding the use of T. durum instead of the poorly assembled taushii: were the mapping stats so bad that the result would be unusable? What about correcting misassemblies in the Aegilops taushii by running 3D-DNA first instead of compromising by using T. durum that may bear some true rearrangements? Alterantively, there is a recent work by Zhou et al., 2021 who provided high-quality references which could be also used.

Response: The genome of T. durum is AABB. We mapped OCEAN-C data of Ae. tauschii to D subgenomes of T. aestivum (AABBDD) rather than T. durum genome. As suggested, we mapped OCEAN-C reads of Ae. taushii to different genomes including the references assembled by Zhou et al. 2021 (AY17, AY61, T093 and XJ02) (Table R2) [5]. When D subgenome of hexaploid T. aestivum as reference, the number of uniquely mapped reads was highest (Table R2) and anomalous chromatin interaction structures in matrix was least (Figure R2). So we still decided to use subgenomes (AABB and DD) of T. aestivum as reference genomes to map OCEAN-C data of the T. durum and Ae. tauschii

As this point has not been raised by the other reviewer, I leave the judgement to the reader, provided that the Table R2 and Figure R2 be included as the Supplemental.

**Authors Response**

**Point-by-point responses to the reviewers’ comments:**

Reviewer #2:

The only point that was not responded to my satisfaction is #6. As this point has not been raised by the other reviewer, I leave the judgement to the reader, provided that the Table R2 and Figure R2 be included as the Supplemental.

*Response: The Table R2 and Figure R2 in previous response letter have been included in the revision as Table S7 and Figure S6. Meanwhile, we addressed all remaining editorial concerns.*
